# Optimal Uses of Antiretrovirals for Prevention in HIV-1 Serodiscordant Heterosexual Couples in South Africa: A Modelling Study

**DOI:** 10.1371/journal.pmed.1001123

**Published:** 2011-11-15

**Authors:** Timothy B. Hallett, Jared M. Baeten, Renee Heffron, Ruanne Barnabas, Guy de Bruyn, Íde Cremin, Sinead Delany, Geoffrey P. Garnett, Glenda Gray, Leigh Johnson, James McIntyre, Helen Rees, Connie Celum

**Affiliations:** 1 Department of Infectious Disease Epidemiology, Imperial College London, United Kingdom; 2 Department of Global Health, University of Washington, Seattle, United States of America; 3 Department of Medicine, University of Washington, Seattle, United States of America; 4 Department of Epidemiology, University of Washington, Seattle, United States of America; 5 Perinatal HIV Research Unit, University of Witwatersrand, Johannesburg, South Africa; 6 Reproductive Health Research Unit, University of Witwatersrand, Johannesburg, South Africa; 7 Centre for Infectious Disease Epidemiology and Research, University of Cape Town, Cape Town, South Africa; Harvard School of Public Health, United States of America

## Abstract

**Background:**

Antiretrovirals have substantial promise for HIV-1 prevention, either as antiretroviral treatment (ART) for HIV-1–infected persons to reduce infectiousness, or as pre-exposure prophylaxis (PrEP) for HIV-1–uninfected persons to reduce the possibility of infection with HIV-1. HIV-1 serodiscordant couples in long-term partnerships (one member is infected and the other is uninfected) are a priority for prevention interventions. Earlier ART and PrEP might both reduce HIV-1 transmission in this group, but the merits and synergies of these different approaches have not been analyzed.

**Methods and Findings:**

We constructed a mathematical model to examine the impact and cost-effectiveness of different strategies, including earlier initiation of ART and/or PrEP, for HIV-1 prevention for serodiscordant couples. Although the cost of PrEP is high, the cost per infection averted is significantly offset by future savings in lifelong treatment, especially among couples with multiple partners, low condom use, and a high risk of transmission. In some situations, highly effective PrEP could be cost-saving overall. To keep couples alive and without a new infection, providing PrEP to the uninfected partner could be at least as cost-effective as initiating ART earlier in the infected partner, if the annual cost of PrEP is <40% of the annual cost of ART and PrEP is >70% effective.

**Conclusions:**

Strategic use of PrEP and ART could substantially and cost-effectively reduce HIV-1 transmission in HIV-1 serodiscordant couples. New and forthcoming data on the efficacy of PrEP, the cost of delivery of ART and PrEP, and couples behaviours and preferences will be critical for optimizing the use of antiretrovirals for HIV-1 prevention.

*Please see later in the article for the Editors' Summary*

## Abstract 2

Hallett et al use a mathematical model to examine the long-term impact and cost-effectiveness of different pre-exposure prophylaxis (PrEP) strategies for HIV prevention in serodiscordant couples.

## Editors' Summary

**Background:**

Every year, about 2.5 million people become infected with HIV, the virus that causes AIDS. HIV is usually transmitted through unprotected sex with an HIV-infected partner. It destroys immune system cells (including CD4 cells, a type of lymphocyte), leaving infected individuals susceptible to other infections. There is no cure for AIDS, although HIV can be held in check with antiretroviral therapy (ART), and there is no vaccine that protects against HIV infection. So, to halt the AIDS epidemic, other ways of preventing the spread of HIV are needed. Antiretroviral drugs could potentially be used in two ways to reduce HIV transmission. First, ART could be given to HIV-infected people before they need it for their own health to reduce their infectiousness; the World Health Organization currently recommends that HIV-positive people initiate ART when their CD4 count drops below 350 cells/µl blood but in many African countries ART is only initiated when CD4 counts fall below 200 cells/µl. Second, ART could be given to HIV-uninfected people to reduce acquisition of the virus. This approach—preexposure prophylaxis (PrEP)—has provided protection against HIV transmission in some but not all clinical trials.

**Why Was This Study Done?:**

Couples in long-term relationships where one partner is HIV-positive and the other is HIV-negative (HIV serodiscordant couples) are a priority group for prevention interventions. In sub-Saharan Africa, where most new HIV infections occur, 10%–20% of stable partnerships are serodiscordant and condom use is often low, not least because such couples may want children. Earlier ART or PrEP might reduce HIV transmission in this group but the merits of different approaches have not been analyzed. In this study, the researchers use a mathematical model to examine the long-term impact and cost-effectiveness of different PrEP and ART strategies for HIV prevention in serodiscordant couples.

**What Did the Researchers Do and Find?:**

The researchers constructed a model to simulate HIV infection and disease progression among hypothetical HIV serodiscordant stable heterosexual couples. The model incorporated data from South Africa on couple characteristics, disease progression, ART use, pregnancies, frequency of sex, and contact with other sexual partners, as well as estimates of the effectiveness of PrEP from clinical trials. The researchers used the model to compare the impact on HIV transmission, survival and quality of life, and the cost-effectiveness of no PrEP with four PrEP strategies—always use PrEP after diagnosis of HIV serodiscordancy, use PrEP up to and for a year after ART initiation by the HIV-infected partner (at a CD4 count of ≤200 cells/µl or ≤350 cells/µl), use PrEP only up to ART initiation by the infected partner, and use PrEP only while trying for a baby and during pregnancy. The model predicts, for example, that the cost per infection averted of PrEP used before ART initiation will be offset by future savings in lifelong treatment, particularly among couples with multiple partners, low condom use, and a high risk of transmission. To keep couples alive without the HIV-uninfected partner becoming infected, it could be more cost-effective to provide PrEP to the uninfected partner than to initiate ART earlier in the infected partner, provided the annual cost of PrEP is less than 40% of the annual cost of ART and PrEP is more than 70% effective. Finally, if PREP is 30%–60% effective, the most cost-effective strategy for couples could be to use PrEP in the uninfected partner prior to ART initiation in the infected partner at a CD4 count ≤350 cells/µl.

**What Do These Findings Mean?:**

These findings suggest that the strategic use of PrEP and ART could cost-effectively reduce HIV transmission in HIV serodiscordant stable heterosexual couples in sub-Saharan Africa. The accuracy of these findings depends on the assumptions included in the mathematical model and on the data fed into it. In particular, the interpretation of these results is complicated by uncertainties in the likely cost of PrEP and the “real-world” effectiveness of PrEP. Nevertheless, these findings suggest that PrEP may become a valuable addition in some settings to existing approaches for HIV prevention such as condom promotion and male circumcision programs. Moreover, additional simulations with this mathematical model using more accurate information on the costs and effectiveness of PrEP could assist in future policy making decisions.

**Additional Information:**

Please access these Web sites via the online version of this summary at http://dx.doi.org/10.1371/journal.pmed.1001123.

- Information is available from the US National Institute of Allergy and infectious diseases on HIV infection and AIDS
- NAM/aidsmap provides basic information about HIV/AIDS, summaries of recent research findings on HIV care and treatment, and a section on PrEP
- Information is available from Avert, an international AIDS charity on many aspects of HIV/AIDS, including information on all aspects of HIV prevention, and on HIV/AIDS in Africa (in English and Spanish)
- AVAC Global Advocacy for HIV Prevention provides up-to-date information on all aspects of HIV prevention, including PrEP
- The US Centers for Disease Control and Prevention also has information on PrEP
- WHO provides information about antiretroviral therapy
- Patient stories about living with HIV/AIDS are available through Avert and through the charity website Healthtalkonline

## Introduction

Thirty years after HIV-1 was first recognized, the epidemic continues with 2.6 million people newly infected in the past year [1]. Antiretroviral therapy (ART) can dramatically improve the survival of HIV-1–infected persons and is the cornerstone of strategies to prevent vertical HIV-1 transmission [2],[3]. 5.2 million people living with HIV-1 in low- and middle-income countries have been provided with life-saving ART [1]; however, reduced funding for AIDS programs as a result of the global economic crisis threatens these acheivements, and a sustainable response to the HIV-1 epidemic requires a large reduction in the numbers becoming infected [1],[4],[5].

Many different forms of interventions are used to help reduce the spread of HIV, and recently UNAIDS have proposed a framework that prioritises condom promotion, interventions for key populations, behaviour change programmes, male circumcision, prevention of mother-to-child transmssion, and treatment for people living with HIV as a basic set of program activities that should form the core of responses to the epidemic [6]. Recently, substantial scientific interest has developed in antiretroviral-based strategies for prevention of sexual HIV-1 transmission [7],[8]. In the past year, four clinical trials have closed confirming that antiretrovirals have the potential to be used as: (i) ART to reduce the infectiousness of HIV-1–infected persons [9], and (ii) oral or topical pre-exposure prophylaxis (PrEP) for uninfected persons to reduce acquisition [10]–[12]. Although there is much scientific enthusiasm about antiretroviral-based HIV-1 prevention, many questions remain about how best to marshal these tools to achieve the optimal impact [13],[14]. Here we aim to synthesize these new findings to understand how PrEP and ART could be used to reduce HIV transmission in stable HIV serodiscordant couples.

ART reduces plasma and genital HIV-1 concentrations to undetectable levels in most treated individuals [15],[16], and observational studies, and a recent large multicentre clinical trial, have demonstrated large reductions in HIV-1 infectiousness (>90% reduction in transmission risk) in persons receiving ART [9],[17]–[21]. WHO guidelines currently recommend ART initiation at CD4 counts of <350 cells/µl, although guidelines in many African countries delay ART until CD4 counts decline to <200 cells/µl because of constrained resources [22]. Apart from the initial peak in viral load at seroconversion (during which substantial proportions of transmission events could occur [23],[24]), the average per-contact risk of HIV-1 transmission is highest from those with lower CD4 counts (i.e., below 200 cells/µl [20],[25]), and treatment could potentially reduce transmission from those individuals. However, individuals can be infectious for many years before the CD4 cell count declines to <200 cells/µl or clinical disease necessitates ART, allowing for substantial potential of HIV-1 transmission before treatment is started. Earlier ART initiation, at higher CD4 counts, has therefore been proposed as one HIV-1 prevention strategy [26],[27].

A recent clinical trial of the pericoital use of topical 1% tenofovir gel among HIV-1–uninfected women [10] demonstrated a significant reduction in the risk of HIV-1 acquisition, by approximately 40% overall and by >50% among those with high adherence to the intervention. Two further clinical trials testing the efficacy of daily oral tenofovir and oral emtricitabine-tenofovir in serodiscordant couples and heterosexual women have recently stopped, reporting a strong protective effect (>60%) of PrEP [11],[12]. These findings mirror those for daily oral PrEP in men who have sex with men (the iPrEX study) [28]. One trial (FemPrEP) testing the effect of oral daily emtricitabine tenofovir PrEP in heterosexual women [29] and the part of a large trial (the VOICE study) testing the effect of oral daily tenofovir in women [30] have also been stopped after interim reviews found that it was unlikely these studies would demonstrate benefit, and investigations in the likely causes for this are underway.

High enthusiasm for antiretroviral-based HIV-1 prevention has been balanced by recognition of the need for strategies to efficiently deliver these expensive new prevention options [8]. It may be optimal and cost-effective in concentrated epidemics to preferentially deliver these interventions to those at highest risk of transmission, such as sex workers or individuals that inject drugs if benefit is observed on on-going trials [31]. However, in the generalized heterosexual epidemics in sub-Saharan Africa, most transmission occurs outside of such easily identified risk groups [1]. One group that has been identified as a high priority for HIV-1 prevention is uninfected individuals in long-term sexual partnerships with HIV-1–infected individuals, i.e., stable HIV-1 serodiscordant couples [32]–[35]. The proportion of stable partnerships in southern Africa that are serodiscordant has been estimated to be between 10% and 20% [36], and in the community mobilization work for the Partners in Prevention Study, 27% of couples tested at three South African sites (Cape Town, Orange Farm, and Soweto) were HIV-1 serodiscordant [37]. Condom use is typically low in stable partnerships [38], particularly during periods when couples desire pregnancy, during which the risk of HIV-1 acquisition and transmission is increased [39]. HIV-1 incidence can be high in these partnerships and, importantly, not all of the risk to the uninfected individual comes from their stable partner. By comparing the genetic sequence of the virus in serodiscordant couples where the HIV-1–uninfected partner became infected, it has been estimated that the HIV-1–infected partner was not the source of infection in approximately ∼30% of couples [40]. In these cases, PrEP—but not ART for the infected partner—could offer protection against infections that might arise from additional partners.

Given the constrained resources for HIV-1 treatment and prevention in sub-Saharan Africa, many questions need to be considered regarding the relative benefits and costs of PrEP and earlier ART for HIV-1 prevention, specifically in potential target groups such as HIV-1 serodiscordant couples, including: (i) Is there any benefit of PrEP when ART is available and initiated promptly upon meeting CD4 criteria for the HIV-1–infected partner and, if there is, what patterns of PrEP use maximize this benefit?; (ii) when might earlier ART initiation (at either CD4 cell count <350 or <500 cells/µl) in the HIV-1–infected partner be a more effective use of resources than providing PrEP to the uninfected partner?; and (iii) what is the best way to combine use of PrEP and ART to maximize impact and efficiency? To address each of these questions, we constructed an individual-based model describing HIV-1 serodiscordant couples in South Africa in which the use of PrEP and ART can be simulated and the effects on transmission and life-years saved can be quantified.

## Methods

### Model Structure and Parameterisation

We constructed a microsimulation model of HIV-1 disease progression, transmission of infection, and treatment in stable HIV-1 serodiscordant heterosexual couples in South Africa. The model included the composition of couples (by sex, age, and current CD4 cell counts), ageing, disease progression, use of ART, conception and pregnancies, variations in coital frequency within stable partnerships and contact with other sexual partners. The model tracks each individuals' trajectories over time until they die. Each result presented is the mean for sets of 20,000 simulated couples. Key assumptions are summarized in Table 1 and a full description and all parameter values are provided in Text S1. The model was initially parameterised using data collected at three South African sites (Cape Town, Orange Farm, and Soweto) that participated in the Partners in Prevention HSV/HIV Transmission Study, a multinational prospective HIV-1 prevention clinical trial among HIV-1 serodiscordant couples [41],[42] in which the HIV-1–infected partner had CD4 >250 cells/µl and was not eligible for ART by national guidelines; we refer to this assumption as the “partners in prevention” scenario. HIV-1 seroincidence was relatively low (∼1.8/100 person-years at risk [pyar]) in the Partners in Prevention HSV/HIV Transmission Study cohort, which was likely owing to selecting couples willing to participate in an HIV-1 prevention clinical trial, frequent couples risk-reduction counselling, and resultant high condom use. Therefore, we constructed another set of assumptions, named “more typical couples”, in which 50% of the partnerships involved HIV-1–infected men (to correspond to the gender distribution of HIV-1 serodiscordancy in population-based studies in Africa [34]), condom use within the stable partnership was reduced to 75% of that reported in the partners in prevention cohort, 50% more of the HIV-1–uninfected partners in couples had external partners, and the frequency of unprotected sex with external partners was double that reported in the Partners in Prevention Study [41],[42]. The assumptions made in the “more typical couples” scenario were such that the overall incidence rate among these couples was consistent with other empirical measurements of HIV-1 incidence in serodiscordant couples (7.7/100 pyar in Zambia [43] and 9.2/100 pyar in Uganda [21]) and reflected the balance of infections from stable partners versus other partners implied by phylogenetic analysis of viral sequences in couples in Rakai, Uganda [44]. Three other sets of assumptions about couples' behaviour were also made and the results are shown in the supplementary materials (Text S1).

**Table 1:** Key assumptions made in the model.

Parameter	Values	Source
Infectiousness of untreated individuals (relative to those with CD4 cell count ≥500 cells/µl)	CD4 350–500: 1.00	Cohort of stable serodiscordant couples [20]
	CD4 200–350: 1.59	
	CD4 0–200: 4.99	
Mean time spent in CD4 cell count category (y)^a^	Infection to CD4 of 500: 2.4	Pooled analysis of African observational cohort studies [66]
	CD4 350–500: 2.4	
	CD4 200–350: 4.6	
	CD4 0–200: 2.6	
Relative infectiousness of those on ART (relative to those untreated with CD4 cell count <350 cells/µl)	0.08	Cohorts of stable serodiscordant couples [17],[20]
Mortality rates on ART (per year)		Multinational observational cohort studies [67]–[69]
First year:		
ART initiation at CD4 500+:	1.3%	
ART initiation at CD4 350–500:	2.5%	
ART initiation at CD4 200–350:	5%	
ART initiation at CD4 0–200:	10%	
Subsequent years:		
ART initiation at CD4 500+:	1.3%	
ART initiation at CD4 350–500:	1.3%	
ART initiation at CD4 200–350:	2.5%	
ART initiation at CD4 0–200:	5%	
Drop-out from ART (per year)	First year: 10%; subsequent years: 5%	Observational data from programs in Zambia [70]
PrEP effectiveness	30%–80% (in Figure 1)	Consistent with the ranges of effectiveness reported in a large trial of PrEP in serodiscordant couples [11]
Full cost per year of ART	US$450–US$800 (midpoint: US$625)	[45]–[47]
Full cost per year of PrEP	US$150 and US$250 (midpoint: US$200)	[53]
Relative annual cost of PrEP compared to ART	18%–56% (midpoint: 32%)	Calculated from values given above

a Mean time elapsed between entering category (CD4 cell count reaching value of upper bound) and exiting category (CD4 cell count drops below value of lower bound).

The parameter for the “effectiveness” of PrEP, which combines assumptions about the “intrinsic efficacy” of PrEP and levels of adherence to the regimen (Text S1), is analogous to the estimates of effect size that have been generated in PrEP clinical trials. In the initial analyses we assumed effectiveness values as high as 80% (the upper limit of the 95% confidence interval for the effect of oral emtricitabine-tenofovir in serodiscordant was 85% [11] and the estimate of effect for men and women receiving tenofovir in another study was 78% [12]) and as low as 30% (the lower limit of the 95% confidence interval for the effect of tenofovir in serodiscordant couples was 34% [11]). As well as the statistical imprecision in the effect size found in the trials, this range also reflects that the effectiveness of PrEP in “real-world” roll-out may not reproduce that found in clinical trials.

The cost of one person-year of PrEP was assumed to range between US$150 and US$250 on average (this includes lab testing, personnel, and drug costs), and the approximate costs of each additional person-year of ART was set at US$450–US$800 [45]–[47]. This assumption gives a range for the PrEP costs per year as a fraction of the ART costs per year of 18% to 56%. All cost calculations incorporate an annual discount rate of 3%.

### Analysis

The model was analyzed by running a “baseline” scenario in which the only intervention was the initiation of ART for the infected partner, with initiation occurring when their CD4 cell count fell below 200 cells/µl (which until recently was the South African guideline for asymptomatic or nonpregnant HIV-infected individuals [48] and other settings) or 350 cells/µl (assuming adoption of the revised WHO ART initiation guidelines [22]). Relative to this baseline scenario, the impact of particular PrEP and/or ART interventions on three outcomes was quantified: (i) HIV-1 infections averted, which represents the reduction in the number of couples in which the initially HIV-1–uninfected partner is infected before their 50th birthday; (ii) being “alive and HIV-1 free at age 50,” which represents the increase in couples whose HIV-1–uninfected partner is uninfected at his/her 50th birthday and both members of the couple survive to their 50th birthdays; (iii) quality-adjusted life-years (QALYs) saved, which represents the total extra QALYs accrued by the couple because of the intervention. The person-years lived by each partner in the couple are weighted according to the utility associated with each health state (uninfected or infected and according to CD4 cell count category or treatment-status) [49].

These outcomes were chosen to capture the long-term impact on couples, so that the cumulative risk of transmission/death and total costs can be fully reflected. The latter two outcomes record the beneficial effects of these interventions on survival and quality of life, as well as HIV-1 transmission rates.

Three analyses were conducted to address the questions outlined above. In the first analysis, the impact of four different PrEP implementation strategies was examined (Table 2). In a second analysis, one strategy for PrEP use was compared with an alternative intervention of initiating ART for the HIV-1–infected partner earlier than is the current practice in many African countries (i.e., ART initiation at CD4 counts ≤350 cells/µl or ≤500 cells/µl). In a final analysis, an optimal combination of PrEP and ART was analyzed by comparing the cost and efficiency of a range of candidate combination strategies.

**Table 2:** PrEP implementation strategies used in the model.

Strategy	Description
Baseline	No PrEP^a^
I	Always use PrEP after diagnosis of HIV-1 serodiscordancy in couples
II	Use PrEP up to the initiation of ART for the stable HIV-1–infected partner, and during the first year of the partner's ART use^b^, then stop PrEP
III	Use PrEP up to the initiation of the stable HIV-1–infected partner on ART, then stop PrEP
IV	Use PrEP only during periods of trying to conceive a pregnancy^c^ and during pregnancy

In strategies I–III, PrEP is initiated following HIV-1 testing of couples, and, in all strategies, PrEP is stopped immediately if the HIV-1–infected partner dies or the initially HIV-1–uninfected partner becomes HIV-1 infected.

a In this and all other scenarios, it is assumed that ART is initiated promptly when the infected partner's CD4 cell count reaches 200 cells/µl.

b An initial period of continued PrEP is allowed until viral load becomes suppressed after ART initiation in the HIV-1–infected partner. PrEP could reasonably be discontinued after an interval of less than 1 y, or be based on viral load monitoring of the infected partner instead.

c In the model, pregnancies can be preceded by a period of “trying to conceive a pregnancy” during which the frequency of unprotected sex increases (see Text S1).

## Results

### The Additional Impact of Different PrEP Interventions in the Context of Routine ART Initiation

**Figure 1:**
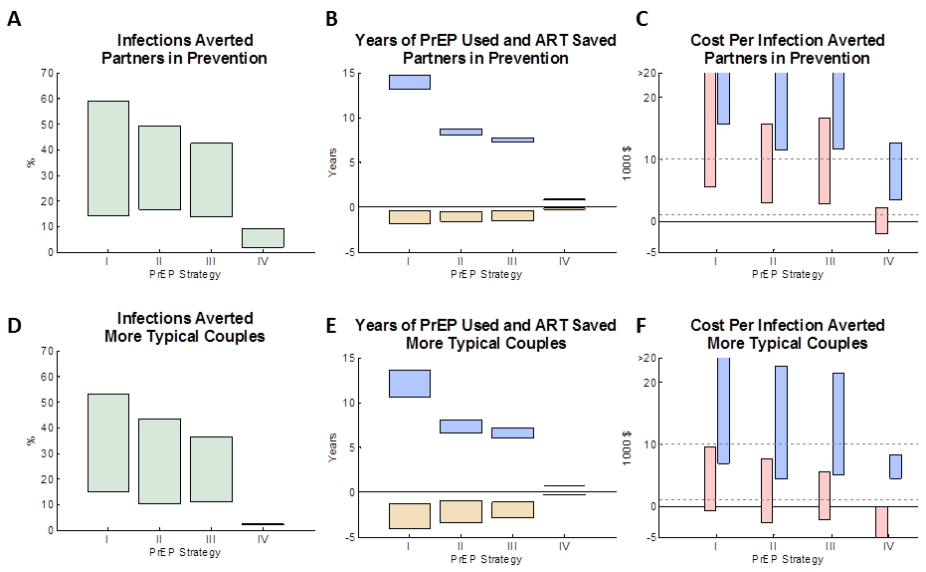
The impact of different PrEP interventions on HIV infections in the couple. (A, D) The proportion of infections averted by age 50 (relative to a baseline strategy with no PrEP intervention) for four PrEP strategies (see Table 2). (B, E) The expected mean years on PrEP (blue boxes) and years on ART averted (yellow boxes) for each of the four PrEP interventions (after discounting). (C, F) The expected cost per infection averted for each of the four PrEP interventions: the pink boxes reflect the lower PrEP cost estimates (and the higher ART cost estimates) and the blue boxes reflect the higher PrEP cost estimates (and the lower of ART cost estimates). In (A–F), the boxes shows a “feasible” range of results, which corresponds to a functional effectiveness of PrEP ranging between 50% and 80%. The assumptions used about the couple's behaviour are: (A–C) “partners in prevention” assumptions and (D–F) the “more typical couples” assumptions (see main text for details). Figure S2 shows the analysis repeated for alternative types of couples. (Summary of strategies from Table 2: I, always PrEP; II, PrEP prior to ART with 1-y overlap; III, PrEP prior to ART (no overlap); IV, PrEP during conception/pregnancy.)

The modelled impact of the four PrEP implementation strategies in the “partners in prevention” couples and “more typical couples” is shown in Figure 1 (bars assume a PrEP effectiveness ranging from 30% to 80%). For both types of couples, the greatest number of infections is averted (14%–59% and 15%–52%, respectively) when PrEP is used at all times, irrespective of ART use by the HIV-1–infected partner (Figure 1A and 1D: strategy I). PrEP has a greater proportionate effect in the “partners in prevention” couples because the overall risk of transmission is lower at baseline. The number of infections averted was somewhat less (14%–43% and 11%–36%) if PrEP was discontinued when the HIV-1–infected partner initiated ART (strategy III) or 1 y afterwards (17%–49% and 10%–43%; strategy II) because of continued HIV-1 risk from outside partners and from ART discontinuation in the HIV-1–infected partners. Because the HIV-1–infected partner's infectiousness decreases gradually after ART is initiated, the estimated number of infections averted was only marginally higher if PrEP is used during the first year of ART (strategy II) rather than discontinuing PrEP in the HIV-1–uninfected partner immediately upon ART initiation by their HIV-1–infected partner (strategy III). Using PrEP only during periods of trying to conceive a pregnancy and during pregnancy (strategy IV) averts only a small proportion of infections overall (2%–10% and 1%–2%), because the time spent protected by PrEP is short relative to the many years the couples spend together. This model does assume, however, that there is some continued risk of transmission of HIV in the couples when they are not trying to conceive, and although this has been observed in cohort studies [41],[42], it is not known if this would be the case for couples seeking to use PrEP in this way.

The four PrEP strategies differed in the amount of PrEP used (Figure 1B and 1E, blue bars). Using PrEP throughout the duration of the partnership (strategy I) resulted in an average of ∼15 y (“partners in prevention”) or ∼12 y (“more typical couples”) of PrEP use per couple, whilst only using PrEP up to the initiation of treatment (strategy III) uses ∼7–8 y of PrEP per couple, and only using PrEP during conception and pregnancy (strategy IV) uses less than 1 y of PrEP per couple. The PrEP intervention strategy also influences the total amount of ART used by the couple, because when an infection is averted the protected individual will not later require ART (Figure 1B and 1E, yellow bars). The average amount of ART saved (yellow bars) correlates with the average number of infections averted, with up to 2 y (“partners in prevention”) or 4 y (“more typical couples”) of ART potentially saved if PrEP were to be used throughout the partnership (strategy I). In comparison, strategies II and III used substantially less PrEP while saving less total ART.

Costs per infection averted were calculated taking into account the lifetime costs of PrEP and ART for the initially uninfected individual who seroconverts. In the “partners in prevention” couples (Figure 1C), under strategy I (when PrEP is always used) each infection averted costs between US$6,000 and US$66,000 (depending on the effectiveness and the cost per year of PrEP). We note that these costs are lower than the “up-front” only cost of PrEP (US$11,000–US$69,000 per infection averted because the total costs also reflect the eventual ART savings that accrue for each infection averted. The most cost-effective strategies were strategies II and III (using PrEP prior to ART initiation by the HIV-1–infected partner or up until 1 y after ART initiation), which gave a minimum expected cost of US$3,000 per infection averted, for the model with the highest assumed effectiveness (80%), lowest assumed PrEP cost, and highest assumed ART cost. This result can also been seen when comparing the incremental cost and impact of PrEP in averting infections (Figure S1) between the PrEP strategies: the incremental cost-effectiveness of using PrEP prior to ART initiation (scenario II/III) compared to not using PrEP (baseline scenario) is high, whereas the marginal benefits of additionally using PrEP whilst their partner is on ART (scenario I) is small relative to the extra cost. Because scenarios II and III give similar costs and benefits (especially when compared with the other scenarios simulated: see Figure S1 and the overlap of confidence intervals), and because any decision to cease PrEP use at the time of a partner's ART initiation may also be informed by monitoring clinical symptoms, CD4 cell count, and viral load (rather than simply time elapsed since initiation), no further analyses are conducted to differentiate these two scenarios.

In the “more typical couples”, costs per infection averted were much lower (Figure 1F), because increased sex in the couple and more external partners results in more infections in the absence of PrEP, and so a greater number of infections can be averted. With these assumptions, the net cost per infection averted under strategy I ranged between ∼US$0 (“break-even”) and US$26,000, and, under strategy III, between US$–2,200 (net cost-saving) and US$21,000. The estimated cost per infection averted when using PrEP only during conception or pregnancy (strategy IV) was highly variable in simulations (due to the relatively rare occurrence of pregnancy in uninfected partners in this situation prior to their partner starting ART), but overall the model indicated that this strategy could be a cost-effective way to use PrEP (cost per infection range was US$–2,000 to US$12,000 for “partners in prevention” and US$–6,000 to US$8000 for “more typical couples”).

For scenario III, the corresponding estimates of the costs per QALY saved are: for 30% effective PrEP, US$2,500–US$4,900 per QALY in “partners in prevention” couples or US$700–US$1,900 per QALY in “more typical couples” (depending on cost of PrEP); and for 80% effective PrEP, US$260–US$1,600 per QALY in “partners in prevention” couples or US$–200 (cost-saving) to US$500 per QALY in “more typical couples”. This result places PrEP among the more expensive public health interventions [50],[51], although it does likely meet the thresholds for costs per life-year saved suggested by WHO for the region [52].

The analyses were repeated in three other sets of couples with different types of behaviours (Figure S2). Compared to the “partners in prevention” assumptions, the cost per infection averted was lowest if it was assumed that condoms were used less frequently by the couple, because more transmissions occur prior to the HIV-1–infected partner's initiation of ART. For the “more typical couples” with more external partners, there was a greater relative advantage in using PrEP regardless of the known HIV-1–infected partner's ART use, because PrEP will reduce the risk of HIV-1 acquisition from all partners, whereas ART for the stable HIV-1–infected partner only reduces the infectiousness and likelihood of transmission from their known HIV-1–infected partner. If a higher proportion of men were assumed to be initially infected, the results were similar to those in the models with “partners in prevention” behaviour assumptions.

### Comparison of PrEP and Earlier ART Initiation

**Figure 2:**
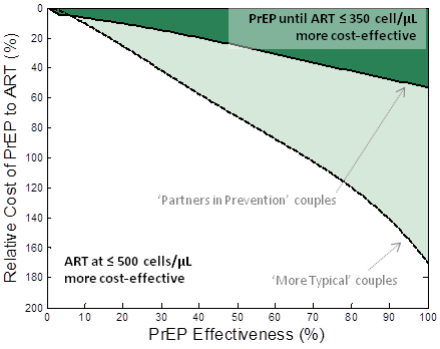
Comparison of PrEP versus earlier ART initiation for keeping couples “alive and HIV free at 50.” The relative cost of PrEP to ART (vertical axis) and the effectiveness of PrEP (horizontal axis) are varied and the shaded region indicates the conditions where a PrEP intervention (PrEP used up to the moment that their infected partner starts treatment (at CD4<350 cells/µl)) is at least as cost-effective as earlier initiation of ART (at CD4<500 cells/µl) at allowing couples to be “alive and HIV-1 free at 50.” The dark shaded region corresponds to the “partners in prevention” assumptions about couples' behaviour and the lighter shaded region corresponds to “more typical couples” behaviour assumptions. Alternative analyses are presented where different assumptions about ART initiation and couples' behaviour are made (Figure S3) and where the comparison is based on savings of QALYs (Figure S4).

We performed a comparison between an intervention that provides PrEP to the HIV-1–uninfected partner until the HIV-1–infected partner initiates ART at CD4<350 cells/µl (strategy III) and an intervention providing earlier ART initiation to the infected partner at a CD4 cell count of 500 cells/µl but no PrEP. Both interventions increase cost and reduce infections. The comparison metric was the number of couples “alive and HIV-1 free at age 50.” Figure 2 shows the required properties for PrEP (relative cost and effectiveness) to be at least as cost-effective as earlier ART initiation under these assumptions. With the assumed behaviours observed among the “partners in prevention” couples, PrEP would be at least as cost-effective as earlier ART if its effectiveness was more than ∼75% and the annual cost was less than 40% that of ART (Figure 2, dark shaded area). At the upper estimate of the relative annual cost of PrEP (56% of ART costs [53]), PrEP effectiveness would need to be more than 90% to be at least as cost-effective as earlier ART initiation at ≤500 cells/µl in these couples.

A much broader range of PrEP effectiveness and cost values render PrEP at least as cost-effective as earlier ART if the behaviours characterizing the “more typical couples” are assumed (Figure 2, lighter shaded area). In this case, with the highest current cost estimates, PrEP would be at least as cost-effective as earlier ART initiation at 350 cells/µl if it has effectiveness greater than ∼40%. This result is because, in these couples, there is a high risk of infection before ART is started and a lower proportion of transmissions are from the stable HIV-1–infected partner (thus, reducing the infectiousness of the HIV-1–infected partner using ART in the stable HIV-1–infected partner only partially reduces the risk to the HIV-1–uninfected partner). The analyses are repeated for different assumptions about couples' behaviour in Figure S2.

We repeated these analyses in the context of earlier ART initiation, comparing PrEP use prior to ART initiation at CD4 cell counts below 200 cells/µl with earlier ART initiation at 350 cells/µl. The pattern is similar to the previous analysis and in “more typical couples”, PrEP with effectiveness of 50% could have an annual cost as much as 70% that of ART and be as cost-effective as earlier ART initiation for the HIV-1–infected partners. At the upper-bound of the estimate for relative PrEP-to-ART costs, PrEP would be as cost-effective as initiating ART in the HIV-1–infected partner at CD4<500 cells/µl if PrEP was more than 40% effective.

Both analyses were repeated using the QALY outcome (Figure S3). Here, the requirements for PrEP to be more cost-effective than earlier ART (at either ≤350 or ≤500 cells/µl) become more stringent (although the differences with respect to the alternative types of couples, described above, remain.) For example, for PrEP and ART at 350 cells/µl to be more cost-effective than ART at 500 cells/µl in “more typical couples”, PrEP should have an effectiveness greater than ∼50% if the per-year cost of PrEP was half that of ART, compared to ∼40% using the “alive and HIV-1 free” outcome. This result is because a new infection is a much less severe outcome than a premature death when considering utilities in the QALY calculation, whereas infection and premature death are given equal weight in the “alive and HIV-1 free at 50” outcome.

An additional analysis quantified the influence of the assumed mortality and drop-out rates on infections averted for couples for whom ART is initiated at CD4 cell counts <500 cells/µl instead of CD4<200 cells/µl (Figure S5). With 50% higher drop-out rates and 25% higher mortality rates on ART than previously assumed, a small marginal benefit (in terms of infections averted and life-years for the couple) is maintained with earlier initiation of ART, whereas with correspondingly reduced drop-out and mortality rates, the benefit of earlier ART initiation is greater. Further research into the actual drop-out and mortality rates for those initiated on treatment at high CD4 cell counts will therefore be useful in refining this analysis of the trade-offs between earlier ART and the potential use of PrEP.

### Optimal Combinations of PrEP and ART

To determine the best overall strategies for the use of PrEP and ART among HIV serodiscordant couples, the impact (on couples being “alive and HIV free at 50”) and cost (net lifetime costs of treatment and PrEP to the couple) of the full range of possible different strategies were calculated. The strategies were: ART initiated by the HIV-1–infected partner at 200 cells/µl with no PrEP or with PrEP used by the uninfected partner until their partner initiates ART (with varying degrees of PrEP effectiveness: 30%, 60%, or 80%); with ART initiated at 350 cells/µl with no PrEP or with PrEP used by the uninfected partner until ART initiation by their partner (with the same values for PrEP effectiveness); and ART initiated at CD4 count of 500 cells/µl. The impact of all strategies were compared against no treatment. This information is summarized in “impact versus cost” plots (Figure S6).

For both types of couple modelled, in the absence of the option to use PrEP, earlier treatment initiation (up to treatment at CD4<500) could be the most efficient way, among these options, to spend resources in order to keep couples alive and HIV free (but not necessarily an optimal use of resources across a whole HIV program portfolio), although this would be associated with an increase in costs (additional cost compared to treatment at CD4<200: US$2,700 in “partners in prevention” couples and US$3,700 in “more typical couples”). However, if using PrEP were possible and the effectiveness was greater than 30%–40% (assuming the midpoint to high estimate for PrEP cost) then the overall most effective strategy for HIV prevention in higher risk (“more typical”) couples be to offer PrEP to the uninfected partners prior to their partners' treatment initiation.

## Discussion

The analysis provides three main results. First, PrEP used prior to ART initiation can prevent infections in HIV-1 serodiscordant couples and, although the initial costs are high, they are substantially offset by reduced future ART costs among HIV-1–uninfected partners who remain uninfected. In some circumstances (e.g., with effectiveness of 80% and used in couples that remain at high-risk), PrEP could be cost-saving overall. Second, PrEP in serodiscordant couples could be as cost-effective as earlier initiation of ART (compared to existing practice) if PrEP has a sufficiently high effectiveness (>70%) and low cost of delivery (<40% annual cost of ART). If used in couples that remain at high risk, PrEP could be as cost-effective as earlier ART even if PrEP had effectiveness of ∼40%. Third, in lower risk couples, earlier ART at CD4<500 may be the most cost-effective strategy, but, in couples that remain at high risk, PrEP and ART could be used together (PrEP in the uninfected individual prior to ART initiation for their HIV-1–infected partner) to deliver maximal benefit and best cost-effectiveness. We hope this might inform the choices that will be available for HIV prevention in couples. We note, however, that it is important that many other considerations besides cost-effectiveness should inform decision-making for HIV treatment initiation and provision of PrEP in couples, including equitable access and the preferences of the couples themselves.

The principal determinants of the eventual use of PrEP among stable serodiscordant couples will be PrEP effectiveness, relative costs of PrEP and ART delivery, and couples' sexual behaviour. Using the model, we have defined a “target product profile,” the cost and effectiveness level for PrEP at which its use in a couple would to be at least as cost-effective as starting ART earlier in couples with different patterns of behaviour. The model shows that, if couples risk behaviour is reduced through risk reduction counselling, and becomes more like the behaviours reported by the “partners in prevention” clinical trial couples, then earlier initiation of ART is probably a more cost-effective way to manage infection and prevent HIV-1 infection (i.e., keeping couples “alive and HIV free”), unless PrEP in “real world” settings is at least as effective as indicated in recent trials among couples [11]. However, the model also shows that, in couples with risks similar to those recorded in observational studies (“more typical” behaviour assumptions [21],[43]), with a PrEP effectiveness similar to that observed in recent trials [11], and at a cost of delivery consistent with optimistic forecasts [53], PrEP use among the uninfected partner could be as cost-effective as earlier treatment, and even a cost-saving intervention in its own right. This outcome highlights how the behavioural profile of couples influences the potential utility of PrEP and illustrates the importance of maximizing efficiency by prioritizing interventions for highest risk couples. It also shows the need for further research into the behaviours of those in long-term serodiscordant couples, their responses to the counselling, and their preferences for these different forms of intervention, in order to develop responsive and appropriate programs.

We note that although the feasibility of delivering ART is proven, the feasibility of PrEP is unknown and currently being investigated, so the information available about each option is not equal. Nonetheless, this analysis does support that PrEP could become one reasonable option that couples in this situation can be offered. And greater choices in HIV prevention should be welcomed as this can lead to increased uptake of services and better protection overall.

These calculations should also inform decision making about investment in new technologies—for instance, by setting a limit on the cost for potential future longer-lasting PrEP formulations that may be more effective. All these considerations are, of course, influenced by the estimated cost of ART, which, through renegotiated drug supply contracts and task-shifting in clinics, might be expected to fall considerably in the coming years [54], which would tend to make earlier ART more cost-effective.

We have explored these trade-offs using a detailed mathematical model that is parameterized and calibrated with data from stable serodiscordant heterosexual couples in South Africa, which included information on the sources of infection for those acquiring HIV-1 (i.e., whether infected by their stable partner or another partner). However, these couples may have lower risks of infection than HIV-1 serodiscordant couples in the general population due to study eligibility criteria and their participation in intensive HIV-1 prevention counselling during a clinical trial. Nonetheless, PrEP delivery programs would require initial HIV testing, and ideally will promote and provide couples HIV counselling and testing, so knowledge of serostatus and condom use will likely increase as has been reported among HIV serodiscordant couples in other studies [55],[56]. Sensitivity analyses were used to explore how differences in sexual behaviour affected the results. The data available from the Partners in Prevention trial [41],[42] cannot fully specify the long-term behaviour of couples in the model because couples were only followed for a 2-y period, whereas the model tracks individuals over their adult lifetimes. The use of the extended time-horizon of the simulation enabled the analysis to reflect the cumulative risk of transmission/death and total costs, whereas a short-term approach would not indicate whether infections in couples are averted or just delayed and would not capture the full cost implications of different strategies (e.g., because life-years saved and ART costs may follow many years after initial PrEP costs). The choice of outcome measure depends upon the relative value placed on preventing death and preventing HIV infection. The QALY approach emphasizes reduced deaths whereas the “alive and HIV free” metric gives more weight to HIV infection, which would often be survived with treatment. Giving more weight to averted infections also helps to implicitly reflect reduced risk of onward HIV transmission.

If further data become available about the added clinical benefits to patients of ART initiation at higher CD4 cell counts rather than indicated in current national and international guidelines, then these should be used to update the model and revise this analysis. We also note that in the analysis the wider benefits of the intervention (or the cost of nonintervention), such as increased labour availability and economic growth, are not included in the calculations. Issues regarding the trade-offs between PrEP and ART for immediate clinical need, including the attendant ethical considerations, are important in the wider debate about resource allocation in HIV programs, but were not relevant here because we only investigated use of PrEP in couples after universal access to ART (at current national and international guidelines) has been achieved. Many countries aim to achieve this by 2015 [57], but we recognize that realistically this may not be achieved until many years later [1].

Many simplifying assumptions were made in the model, including not representing any change in risk behaviours during ART or PrEP use (i.e., potential “risk compensation” from feeling less at risk due to PrEP or ART use), the long-term interaction between PrEP and ART effectiveness through selection of resistant strains of virus [58],[59], or potential effects of sexually transmitted infections on the efficacy of ART or PrEP [60]. The model, and the chosen outcome measures, also do not capture the external sexual partner network so that, for instance, it does not account for the possibility that an averted infection terminates a chain of further infections [61], including averted infections among children. This factor may be expected to influence the estimated impact of ART and PrEP similarly (although further work is required to examine this) because, while ART reduces transmission to the infected individual's other partners, PrEP reduces the chance of infection and the subsequent risk of onward transmission to their partners, including during the initial highly infectious phase [25]. The model also does not reflect that the HIV-1 prevalence and infectiousness among external partners will be influenced by patterns of PrEP and ART use in the wider population [62],[63]. The impact of ART on the incidence of other diseases, particularly tuberculosis, was not explicitly captured and this could lead to an underestimation of the benefit of ART, although the CD4-level–specific mortality rates in untreated individuals and utility-weights in the QALY analysis should implicitly reflect the deterioration in health that is associated with advanced HIV-1 infection [64]. Interpretation of the results is further complicated by key uncertainties in the estimates of the cost of PrEP, which is inevitable given that PrEP delivery programs have not yet been implemented. However, the analyses presented here reflect these uncertainties and it is reassuring that our assumptions for the annual cost of PrEP (and the ratio of PrEP to ART costs) are similar to those independently derived by Pretorius et al. [63]. Although these results suggest that the use of PrEP in HIV-1 serodiscordant couples could be cost-effective and have a significant impact on HIV incidence for that group, there are still significant logistical challenges that are not captured in the model. The identification and retention of discordant couples in services varies from setting to setting, and has been shown to be particularly difficult in South Africa. In such settings the feasibility and cost of targeting discordant couples (and, in particular, couples in which the woman might be pregnant/trying to conceive) could make an intervention utilizing PrEP much more expensive. Lastly, although we hope that this model will assist in policy-making decisions, we recognize that other factors beyond effectiveness and cost will also influence the introduction of PrEP for certain groups.

This analyses focuses on heterosexual HIV-1 serodiscordant couples in sub-Saharan Africa but similar questions could be asked for other groups such as men who have sex with men (MSM) in Africa and elsewhere. Different behavioural, biological, and program parameter values would be required for analyses in these different high risk groups reflecting for example the much higher risk of transmission per unprotected sex act [65] in MSM and the higher cost of treatment for MSM in developed countries. However, the same general principles would apply: the lower the cost and the higher the effectiveness of PrEP, the more likely it is that PrEP will be a cost-effective way to support serodiscordant couples.

In summary, PrEP might become a valuable addition to combination approaches for HIV-1 prevention among stable serodiscordant couples in sub-Saharan Africa, in conjunction with ART. If PrEP is used by individuals that remain at high risk of infection prior to a partner's ART initiation, the additional cost per infection averted might be smaller than previously anticipated or the intervention could even be cost-saving, and the use of PrEP could be as cost-effective as earlier ART initiation. However, this outcome completely relies on the delivery cost of PrEP meeting current forecasts, and the “real-world” effectiveness of PrEP in couples being comparable to that found in the clinical trial [11]: if adherence to PrEP outside of trials is lower, or if PrEP is more expensive to deliver than expected, PrEP could be much less cost-effective. It is vital to understand these trade-offs as soon as possible so that programmatic decision making and implementation can quickly proceed.

## Supporting Information

Figure S1 **Incremental costs and impact of different PrEP implementation strategies for (A) “partners in prevention” couples and (B) “more typical couples”.** In (A) and (B), the extra lifetime (discounted) cost of ART and PrEP (horizontal axis) and the numbers of couples in which an HIV infection is averted (by the age of 50) (vertical axis) are compared when PrEP is: (i) not used (baseline simulation, black star); (ii) always, purple polygon); (iii) up to ART initiation of partner and one year more, red polygon; (iv) up to ART initiation of partner, blue polygon (Table 2). The boundaries of the polygon are given by the ranges of PrEP efficacies and costs given in Table 1. The dot indicates the midpoint of the polygon and the black line connects the scenarios with the highest impacts (infections averted) at different levels of cost.
(PDF)

Figure S2 **The impact of different PrEP interventions in each of the three alternative types of couples (less condom use, more external partners, and more infected men) relative to the characteristics of the partners in prevention cohort.** (A, C, E) The proportion of infections averted (relative to a baseline scenario with no PrEP intervention) for each of the four PrEP interventions (see Table 2). (B,D,F) The expected cost per infected averted for each of the four PrEP interventions: the pink and blue boxes reflect the lower and higher of the PrEP cost estimates and the higher and lower of the ART cost estimates, respectively. In (A–F), the boxes show a feasible range of results, which corresponds to a functional efficacy of PrEP ranging between 50% and 80%.
(PDF)

Figure S3 **Comparison of PrEP versus earlier ART initiation.** This is the same analysis as shown in Figure 2 in the main text but with the frontiers shown for each of the five sets of couples assumptions [see Text S1]. In (A) and (B), the relative cost of PrEP to ART and the functional effectiveness of PrEP are varied. (A) The area to the right of the lines demarcates a region where PrEP use prior to partners' treatment at CD4 cell count below 200 would lead to more couples being “alive and HIV Free at 50” than an ART intervention of the same cost whereby the infected partner is initiated on treatment at CD4 cell count below 350. (B) The area to the right of the lines demarcates a region where PrEP use prior to partners' treatment at CD4 cell count below 350 would lead to more couples being “alive and HIV free at 50” than an ART intervention of the same cost whereby the infected partner is initiated on treatment at CD4 cell count below 500. The different lines show the frontier for the following assumptions about couples behaviour: “partners in prevention,” solid black line (as shown in Figure 2); “less condom use,” dashed blue line; “more extra partners,” dashed green line; “more men infected,” solid grey line; and “more typical couples”, dashed pink line (as shown in Figure 2). Cost is calculated as the total lifetime discounted cost of person-years on PrEP and ART of both partners in initially HIV-1 serodiscordant couples.
(PDF)

Figure S4 **Comparison of PrEP versus earlier ART initiation.** This is the same analysis as shown in Figure S2 but with outcome defined as QALYs [see Text S1]. In (A) and (B), the relative cost of PrEP to ART and the functional effectiveness of PrEP are varied. (A) The area to the right of the lines demarks a region where PrEP use prior to partners' treatment at CD4 cell count below 200 would lead to a greater gain in QALYs than an ART intervention of the same cost whereby the infected partner is initiated on treatment at CD4 cell count below 350. (B) The area to the right of the lines demarcates a region where PrEP use prior to partners' treatment at CD4 cell count below 350 would lead to a greater gain in QALYs than an ART intervention of the same cost whereby the infected partner is initiated on treatment at CD4 cell count below 500. The different lines show the frontier for the following assumptions about couples' behaviour: “partners in prevention,” solid black line (as shown in Figure 2); “less condom use,” dashed blue line; “more extra partners,” dashed green line; “more men infected,” solid grey line; and “more typical couples”, dashed pink line (as shown in Figure 2). Cost is calculated as the total lifetime discounted cost of person-years on PrEP and ART of both partners in initially HIV-1 serodiscordant couples.
(PDF)

Figure S5 **The effect of drop-out and mortality assumptions on the impact of ART.** Comparisons of the infections averted in couples (A) and the QALYs accrued by the couple with treatment initiated at CD4<200 (blue bars) or CD4<500 (red bars) making different sets of assumptions about mortality on ART and drop-out from ART. The assumptions about mortality and drop-out from ART are as follows: “default assumptions” uses the parameter values given in Table 1; “lower drop-out and mortality” uses mortality-rates that are 25% lower and drop-out rates that are 50% lower; “higher drop-out and mortality” used mortality-rates that are 25% higher and drop-out rates that are 50% higher.”
(PDF)

Figure S6 **Impact versus costs for combination strategies of ART and PrEP.** (A) “Partners in prevention couples” and (B) “more typical couples.” The strategies depicted are: no intervention, purple star; ART initiated by the HIV-1–infected partner at 200 cells/µl with no PrEP, solid blue triangle or with PrEP used by the uninfected partner until their partner initiates ART (with varying degrees of PrEP effectiveness: open blue circle, 30%; open blue diamond, 60%; or open blue pentagram, 80%); with ART initiated at 350 cells/µl with no PrEP, solid red triangle, or with PrEP used by the uninfected partner until ART initiation by their partner (with the same values for PrEP effectiveness and respective shapes in red); and ART initiated at CD4 count of 500 cells/µl, solid black triangle.
(PDF)

Text S1 **Description of the model structure and all parameter values used in the model with detailed justifications and explanations.**
(PDF)

## Abbreviations

ART: antiretroviral treatment
PrEP: pre-exposure prophylaxis
pyar: person-years at risk
QALY: quality-adjusted life-years
