# Supplementary material for: Optimal Uses of Antiretrovirals for Prevention in HIV-1 Serodiscordant Heterosexual Couples in South Africa: A Modelling Study
Source: PLoS Med. 2011 Nov 15;8(11):e1001123. doi: 10.1371/journal.pmed.1001123 (PMC3217021; doi:10.1371/journal.pmed.1001123)
Supplement: Figure S1 — Incremental costs and impact of different PrEP implementation strategies for (A) “partners in prevention” couples and (B) “more typical couples”. In (A) and (B), the extra lifetime (discounted) cost of ART and PrEP (horizontal axis) and the numbers of couples in which an HIV infection is averted (by the age of 50) (vertical axis) are compared when PrEP is: (i) not used (baseline simulation, black star); (ii) always, purple polygon); (iii) up to ART initiation of partner and one year more, red polygon; (iv) up to ART initiation of partner, blue polygon (Table 2). The boundaries of the polygon are given by the ranges of PrEP efficacies and costs given in Table 1. The dot indicates the midpoint of the polygon and the black line connects the scenarios with the highest impacts (infections averted) at different levels of cost. (PDF) [file pmed.1001123.s001.pdf]

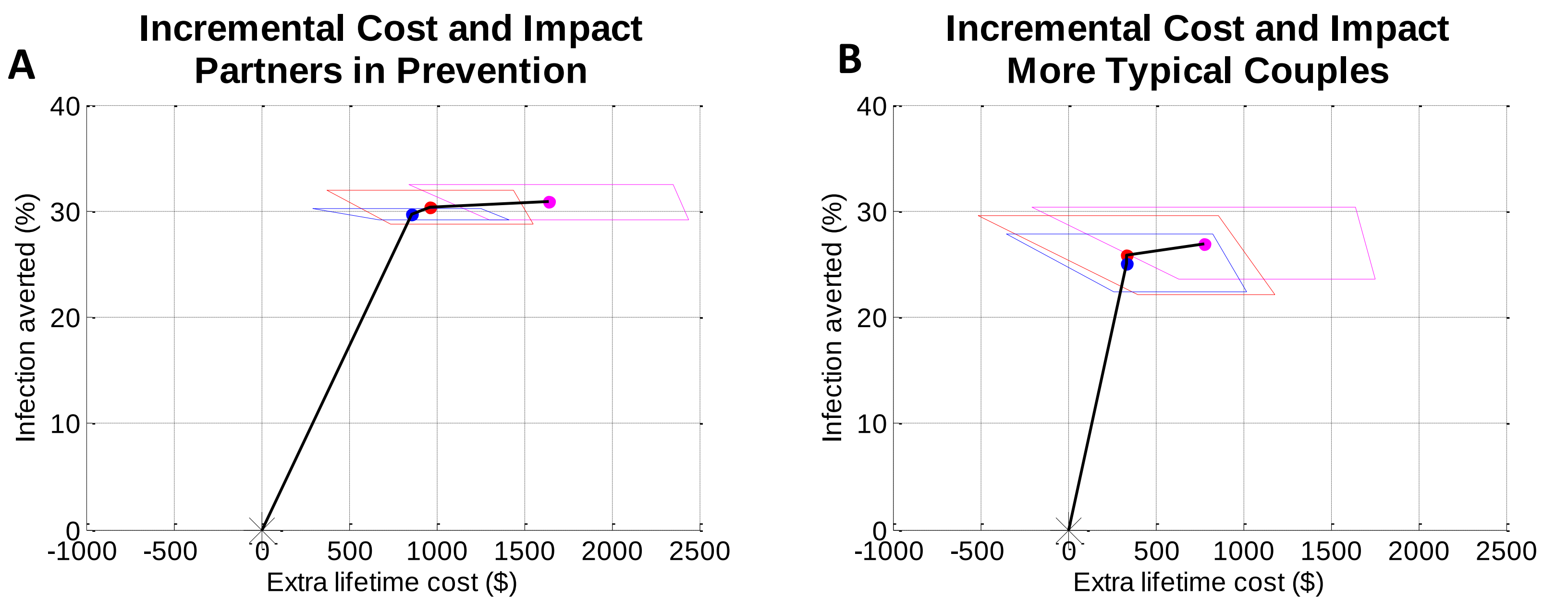

Figure S1: Incremental costs and impact of different PrEP implementation strategies for (A) 'Partners in Prevention 'couples and (B) 'More Typical' couples. In each panel, the extra lifetime (discounted) cost of ART and PrEP (horizontal axis) and the numbers of couples where an HIV infection is averted (by the age of 50) (vertical axis) are compared when PrEP is: (i) not used (baseline simulation: black star); (ii) Always (purple polygon); (iii) Up to ART initiation of partner and one year more (red polygon); (iv) up to ART initiation of partner (blue polygon) (Table 2). The boundaries of the polygon are given by the ranges of PrEP efficacies and costs given in Table 1 . The dot indicates the mid-point of the polygon and the black line connects the scenarios with the highest impacts (infections averted) at different levels of cost.
