# Supplementary material for: Optimal Uses of Antiretrovirals for Prevention in HIV-1 Serodiscordant Heterosexual Couples in South Africa: A Modelling Study
Source: PLoS Med. 2011 Nov 15;8(11):e1001123. doi: 10.1371/journal.pmed.1001123 (PMC3217021; doi:10.1371/journal.pmed.1001123)
Supplement: Figure S2 — The impact of different PrEP interventions in each of the three alternative types of couples (less condom use, more external partners, and more infected men) relative to the characteristics of the partners in prevention cohort. (A, C, E) The proportion of infections averted (relative to a baseline scenario with no PrEP intervention) for each of the four PrEP interventions (see Table 2). (B,D,F) The expected cost per infected averted for each of the four PrEP interventions: the pink and blue boxes reflect the lower and higher of the PrEP cost estimates and the higher and lower of the ART cost estimates, respectively. In (A–F), the boxes show a feasible range of results, which corresponds to a functional efficacy of PrEP ranging between 50% and 80%. (PDF) [file pmed.1001123.s002.pdf]

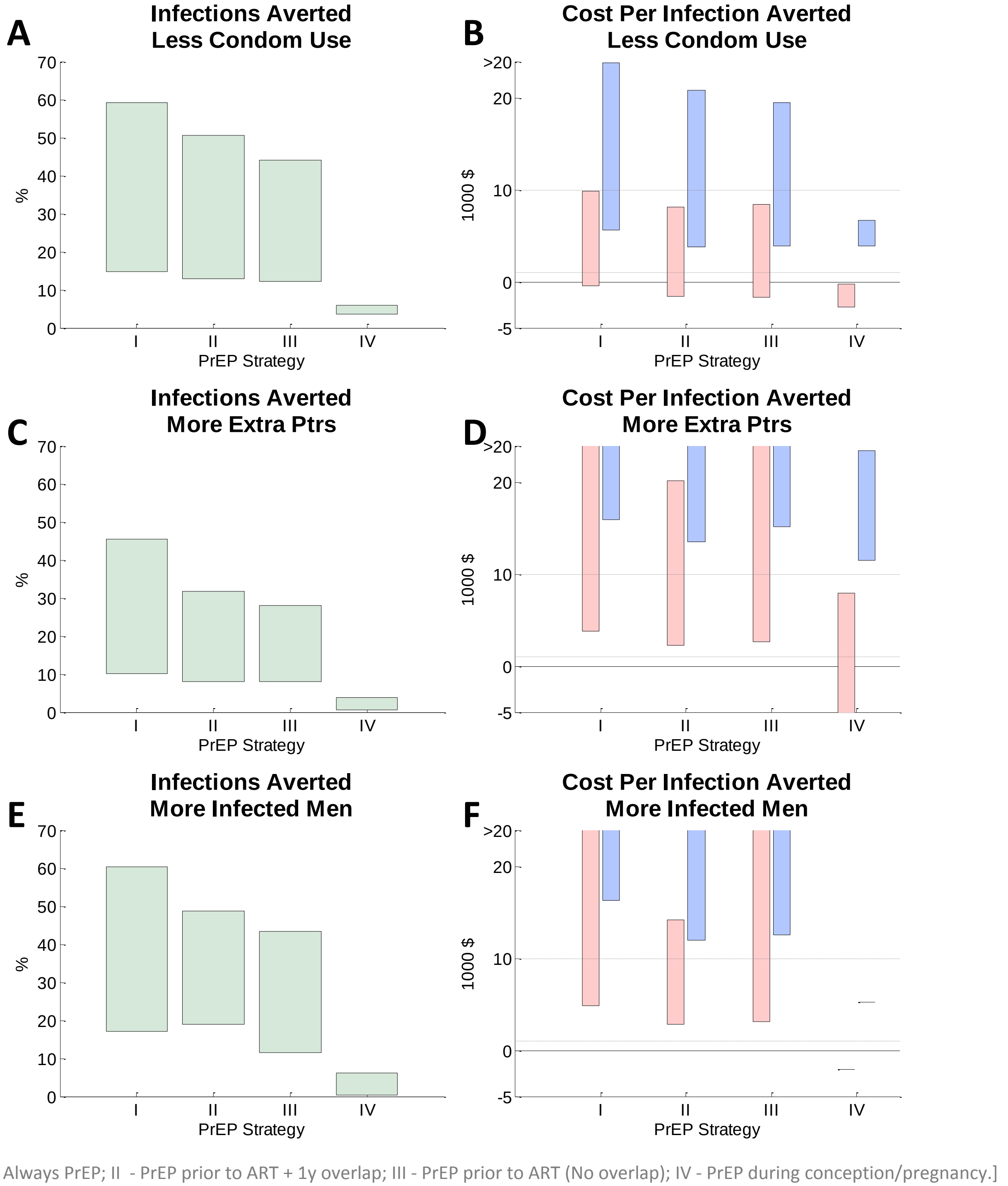

Figure S2: **The Impact of Different PrEP Interventions** in each of the three alternative types of couples (Less Condom Use, More External Partners and More Infected Men) relative to the characteristics of the Partners in Prevention cohort. (A, C , E) The proportion of infections averted (relative to a baseline scenario with no PrEP intervention) for each of the four PrEP interventions (see Table 2). (B,D,F) The expected cost per infected averted for each of the four PrEP interventions: the pink and blue boxes reflect the lower and higher of the PrEP cost estimates and the higher and lower of the ART cost estimates, respectively. In each panel, the boxes shows a feasible range of results, which corresponds to a functional efficacy of PrEP ranging between 50 and 80%.
