# Supplementary material for: Optimal Uses of Antiretrovirals for Prevention in HIV-1 Serodiscordant Heterosexual Couples in South Africa: A Modelling Study
Source: PLoS Med. 2011 Nov 15;8(11):e1001123. doi: 10.1371/journal.pmed.1001123 (PMC3217021; doi:10.1371/journal.pmed.1001123)
Supplement: Figure S4 — Comparison of PrEP versus earlier ART initiation. This is the same analysis as shown in Figure S2 but with outcome defined as QALYs [see Text S1]. In (A) and (B), the relative cost of PrEP to ART and the functional effectiveness of PrEP are varied. (A) The area to the right of the lines demarks a region where PrEP use prior to partners' treatment at CD4 cell count below 200 would lead to a greater gain in QALYs than an ART intervention of the same cost whereby the infected partner is initiated on treatment at CD4 cell count below 350. (B) The area to the right of the lines demarcates a region where PrEP use prior to partners' treatment at CD4 cell count below 350 would lead to a greater gain in QALYs than an ART intervention of the same cost whereby the infected partner is initiated on treatment at CD4 cell count below 500. The different lines show the frontier for the following assumptions about couples' behaviour: “partners in prevention,” solid black line (as shown in Figure 2); “less condom use,” dashed blue line; “more extra partners,” dashed green line; “more men infected,” solid grey line; and “more typical couples”, dashed pink line (as shown in Figure 2). Cost is calculated as the total lifetime discounted cost of person-years on PrEP and ART of both partners in initially HIV-1 serodiscordant couples. (PDF) [file pmed.1001123.s004.pdf]

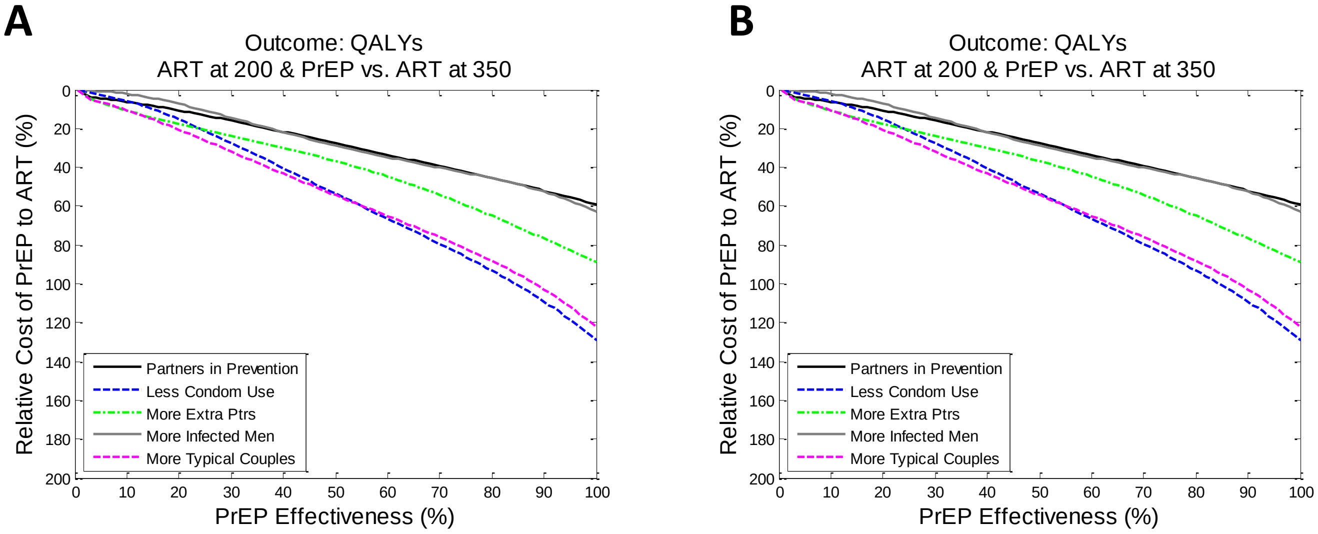

Figure S4: **Comparison of PrEP versus Earlier ART Initiation.** (This is the same analysis as shown in Figure S2 but with outcome defined as Quality-Adjusted Person Years (QALYs). (See Text S1)).

In each panel, the relative cost of PrEP to ART and the functional effectiveness of PrEP are varied. (A) The area to the right of the lines demark a region where PrEP use up prior to partners treatment at CD4 cell count below 200 would lead to a greater gain in QALYs than an ART intervention of the same cost whereby the infected partner is initiated on treatment at CD4 cell count below 350. (B) The area to the right of the lines demark a region where PrEP use up prior to partners treatment at CD4 cell count below 350 would lead to a greater gain in QALYs than an ART intervention of the same cost whereby the infected partner is initiated on treatment at CD4 cell count below 500. The different lines show the frontier for the following assumptions about couples behaviour: 'Partners in Prevention' (solid black line; as shown in Figure 2); 'Less condom use' (dashed blue line); 'More Extra Partners' (dashed green line); 'More Men Infected' (solid grey line); and 'More Typical Couples' (dashed pink line; as shown in Figure 2). Cost is calculated as the total lifetime discounted cost of person-years on PrEP and ART of both partners in initially HIV-1 serodiscordant couples.
