# Supplementary material for: Optimal Uses of Antiretrovirals for Prevention in HIV-1 Serodiscordant Heterosexual Couples in South Africa: A Modelling Study
Source: PLoS Med. 2011 Nov 15;8(11):e1001123. doi: 10.1371/journal.pmed.1001123 (PMC3217021; doi:10.1371/journal.pmed.1001123)
Supplement: Figure S5 — The effect of drop-out and mortality assumptions on the impact of ART. Comparisons of the infections averted in couples (A) and the QALYs accrued by the couple with treatment initiated at CD4<200 (blue bars) or CD4<500 (red bars) making different sets of assumptions about mortality on ART and drop-out from ART. The assumptions about mortality and drop-out from ART are as follows: “default assumptions” uses the parameter values given in Table 1; “lower drop-out and mortality” uses mortality-rates that are 25% lower and drop-out rates that are 50% lower; “higher drop-out and mortality” used mortality-rates that are 25% higher and drop-out rates that are 50% higher.” (PDF) [file pmed.1001123.s005.pdf]

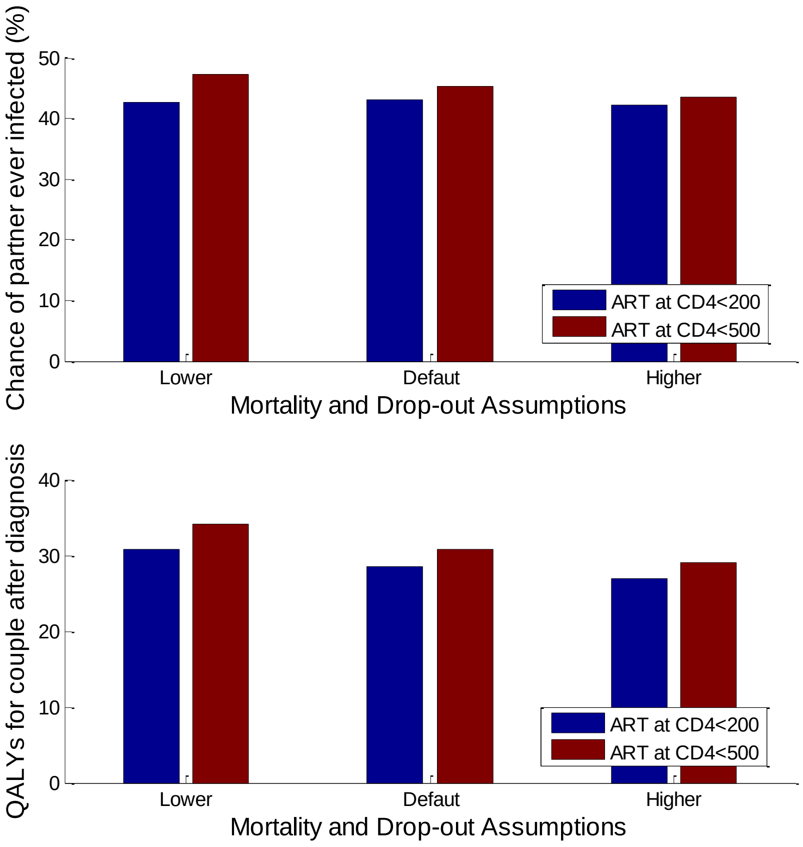

Figure S5: **The effect of drop-out and mortality assumptions on the impact of ART.**
Comparisons of the infections averted in couples (A) and the QALYs accrued by the couple with treatment initiated at CD4<200 (blue bars) or CD4<500 (red bars) making different sets of assumptions about mortality on ART and drop-out from ART. The assumptions about mortality and drop-out on ART are as follows: 'Default Assumptions' uses the parameter values given in Table 1; 'Lower drop-out and mortality' uses mortality-rates that are 25% lower and drop-out rates that are 50% lower'; 'Higher drop-out and mortality' used mortality-rates that are 25% higher and drop-out rates that are 50% higher'.
