# Supplementary material for: Optimal Uses of Antiretrovirals for Prevention in HIV-1 Serodiscordant Heterosexual Couples in South Africa: A Modelling Study
Source: PLoS Med. 2011 Nov 15;8(11):e1001123. doi: 10.1371/journal.pmed.1001123 (PMC3217021; doi:10.1371/journal.pmed.1001123)
Supplement: Figure S6 — Impact versus costs for combination strategies of ART and PrEP. (A) “Partners in prevention couples” and (B) “more typical couples.” The strategies depicted are: no intervention, purple star; ART initiated by the HIV-1–infected partner at 200 cells/µl with no PrEP, solid blue triangle or with PrEP used by the uninfected partner until their partner initiates ART (with varying degrees of PrEP effectiveness: open blue circle, 30%; open blue diamond, 60%; or open blue pentagram, 80%); with ART initiated at 350 cells/µl with no PrEP, solid red triangle, or with PrEP used by the uninfected partner until ART initiation by their partner (with the same values for PrEP effectiveness and respective shapes in red); and ART initiated at CD4 count of 500 cells/µl, solid black triangle. (PDF) [file pmed.1001123.s006.pdf]

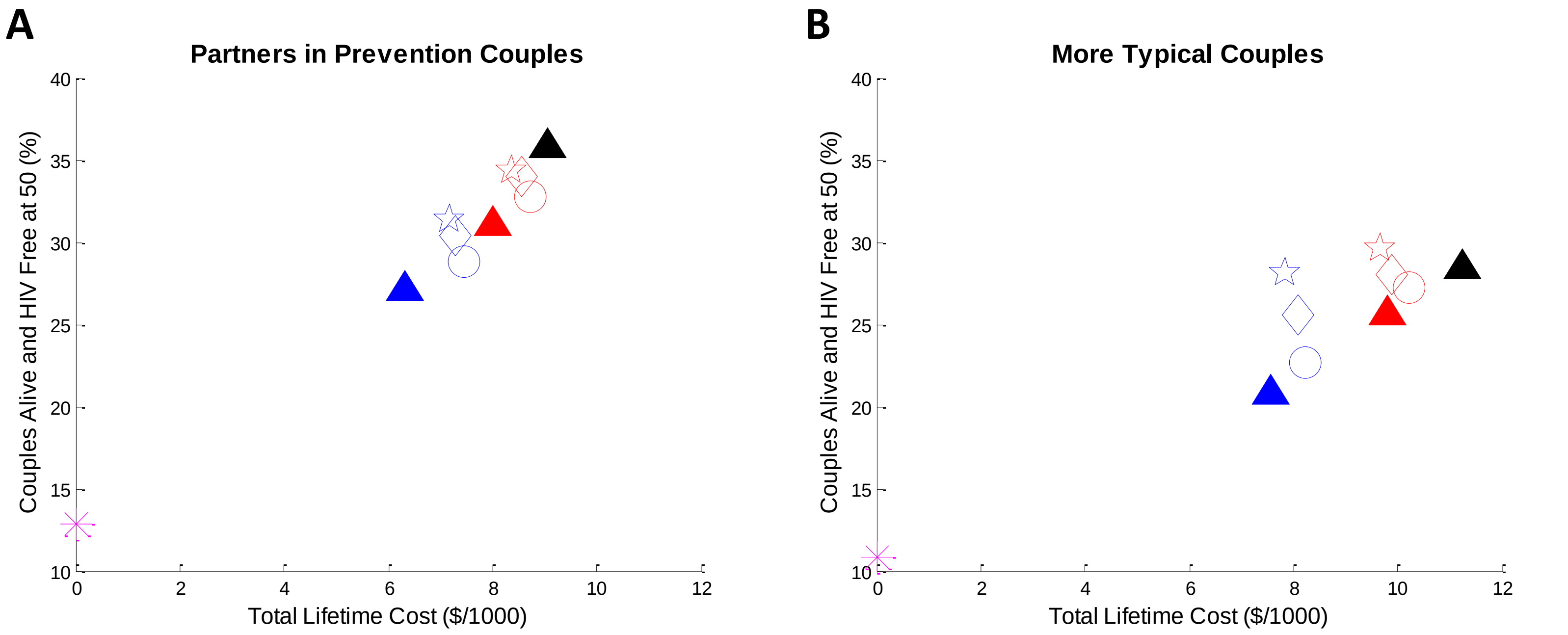

Figure S6: **Impact versus costs for combination strategies of ART and PrEP.**
The panels are for (A) Partners in Prevention Couples and (B) More Typical Couples. The strategies depicted are: No intervention (purple star), ART initiated by the HIV-1 infected partner at 200 cells/µl with no PrEP (solid blue triangle), or with PrEP used by the uninfected partner until their partner initiates ART (with varying degrees of PrEP effectiveness: 30% (open blue circle), 60% (open blue diamond) or 80% (open blue pentagram)); with ART initiated at 350 cells/µl with no PrEP (solid red triangle) or with PrEP used by the uninfected partner until ART initiation by their partner (with the same values for PrEP effectiveness and respective shapes in red); and ART initiated at CD4 count of 500 cells/µl (solid black triangle).
