# Supplementary material for: Optimal Uses of Antiretrovirals for Prevention in HIV-1 Serodiscordant Heterosexual Couples in South Africa: A Modelling Study
Source: PLoS Med. 2011 Nov 15;8(11):e1001123. doi: 10.1371/journal.pmed.1001123 (PMC3217021; doi:10.1371/journal.pmed.1001123)
Supplement: Text S1 — Description of the model structure and all parameter values used in the model with detailed justifications and explanations. (PDF) [file pmed.1001123.s007.pdf]

Text S1

This supplementary text provides an description of the model structure and all parameter values used in the model together with detailed justifications and explanations.

Model Structure

We constructed a detailed individual-based simulation model of HIV disease progression, infection and treatment in stable sero-discordant couples.

The model was programmed in Matlab version 7.11.0[1]. The model starts with couples undergoing HIV testing together and finding that one is infected and the other is not. The infected partner is not indicated for immediate initiation of ART based on current practise in South Africa (i.e. CD4 cell count greater than 200 (1)). Next, the model assigns a sex and age to each of the partners, a certain frequency with which they have unprotected sex, and a CD4 cell count category (200-350, 350-500 or 500+ cell per microlitre) to the infected partner. These characters are drawn at random from the distributions described below, which are based on the Partners in Prevention data (2, 3). The simulation then begins and the couple is tracked until both partners die. Several different events can occur: the infected partner can experience a decline in CD4 cell count, become pregnant, initiate ART or die; the uninfected partner can begin or end sexual partnerships with other individuals, become infected, become pregnant and begin and stop taking PrEP. Periods of 'pregnancy intention' are assumed to precede a fertility event for a set proportion of pregnancies and are characterized by an increase in the average frequency of unprotected sex in the couple.

Transmission within the partnership is a function of the frequency of unprotected sex acts, circumcision status of the male partner, pregnancy status of the female partner, the CD4 cell count/ART-status of the infected partner and the use of PrEP. The influence of sexually-transmitted infections on increasing HIV transmission/susceptibility (4) are not captured in this model. The uninfected partner is also at risk of infection from other sexual partners: a proportion of men and women never have an external partner, while the rest initiate and terminate external partnerships in such a way that the proportion of individuals in external partnerships at any moment matches a specified distribution. The probability that a particular external partner is infected is equal to the

[1] http://www.mathworks.com/products/matlab/

HIV prevalence among the general population of the opposite sex (5). If the partner that was initially uninfected does acquire infection, then the course of their infection is tracked in the same way.

In the model, ART can be initiated at CD4 cell counts of 200, 350 or 500. Over the first 12 months of treatment, the infectiousness of individuals decays exponentially to 8% of the value for those with CD4 cell counts less than 350 (6). Mortality on ART varies by the CD4 cell count at initiation and by the time since initiation (7-9). 'Drop-out' from an ART programme is represented by a hazard of stopping treatment and never starting again (10% per year in the first year of treatment and 5% per year in subsequent years).

The effect of PrEP in the model is to reduce the chance of acquiring infection per sex act with an infected individual by a fixed amount ('intrinsic efficacy'), and the model represents suboptimal adherence to the regimen by only allowing the protective benefit of PrEP in a given proportion of sex acts ('adherence'). These two parameters can be combined to give a functional effect of PrEP (referred to as 'effectiveness' in the main text), which approximates the measurement of effectiveness in a clinical trial; for instance, 80% intrinsic efficacy and 80% adherence generate a 60% functional efficacy, as would be indicated in a trial if there was an incidence rate ratio between intervention and control arms of 0.40 (calculation details below).

Introduction to the Partners in Prevention Data

Between November 2004 and April 2007, 3408 HIV-1 serodiscordant couples from seven African countries (Botswana, Kenya, Rwanda, South Africa, Tanzania, Uganda, Zambia) were enrolled in the Partners in Prevention HSV/HIV Transmission Study, a randomized, placebo-controlled, clinical trial of acyclovir HSV-2 suppressive therapy for the prevention of HIV-1 transmission. Acyclovir did not decrease HIV-1 risk within the couples. This report draws on a secondary analysis of data from this prospective study.

Eligible couples were ≥18 years of age, reported ≥3 episodes of vaginal intercourse during the three months prior to screening, and intended to remain as a couple. HIV-1 infected partners were seropositive for HSV-2, had a CD4 count ≥250 cells/mm3, and were not taking antiretroviral therapy (ART). HIV-1 infected women who were pregnant at screening were excluded from the study and enrolled women who became pregnant during follow-up interrupted study medication use until the completion of pregnancy. Pregnant HIV-1 uninfected women were eligible for enrollment, as they did not receive study medication, and those who became pregnant during follow-up continued usual study procedures.

Participants were followed for up to 24 months, with all participants completing the study by October 2008. HIV-1 infected partners were seen monthly and HIV-1 uninfected partners quarterly. Data on sexual behavior – specifically, number of sexual acts with and without condoms within the partnership and with external partners – were collected at each visit on standardized case report forms using face-to-face interviews in local languages. Plasma for HIV-1 RNA quantification was collected at baseline and months 3, 6, 12, and study exit, and CD4 counts were performed every 6 months. HIV-1 infected persons who met national guidelines for initiation of ART, as a result of CD4 decline or clinical status, were referred to local HIV-1 care clinics. HIV-1 infected women who became pregnant were referred for prevention of mother-to-child transmission services. HIV-1 uninfected partners were seen quarterly for HIV-1 serologic testing. Contraceptive use was recorded at each study visit.

Participants received comprehensive HIV-1 prevention services including HIV-1 risk-reduction counseling (both individual and as a couple), quarterly syndromic sexually transmitted infection (STI) treatment, and provision of free condoms. The study protocol was approved by the University of Washington Human Subjects Review Committee and ethics review boards at each study site. Participants provided written informed consent.

Sex of Uninfected Partner	
Woman	23.5
Man	76.5

Table 1: Sex-distribution of uninfected partners in identified stable sero-discordant partnerships in the default model scenario, based on the Partners in Prevention Data (2, 3). Of the partnerships where the man is uninfected, 56.9% of the men are circumcised and this is also incorporated in the model. Circumcision status of the man does not significantly interact with the distributions of ages of the partners (p=0.119) and so it was assumed that the age-distribution of partners was the same irrespective of circumcision status. In the model, the effect of circumcision is to reduce the probability of infection acquisition per coital act by 65% (10).

**If Man is HIV Negative**									
		**Women's age**							
		15-19	***20-24***	***25-29***	***30-34***	***35-39***	***40-44***	***45-49***	***50+***
**Man's age**	***15-19***	0%	0%	0%	0%	0%	0%	0%	0%
	20-24	1%	4%	2%	1%	1%	0%	0%	0%
	25-29	1%	7%	7%	3%	1%	1%	0%	0%
	30-34	0%	4%	9%	7%	1%	1%	0%	0%
	35-39	0%	3%	5%	7%	2%	1%	1%	0%
	40-44	0%	0%	2%	4%	5%	2%	1%	0%
	45-49	0%	0%	1%	3%	1%	2%	3%	1%
	50+	0%	0%	0%	1%	1%	1%	2%	2%
**If Woman is HIV Negative**									
		**Women's age**							
		**15-19**	**20-24**	**25-29**	**30-34**	**35-39**	**40-44**	**45-49**	**50+**
**Man's age**	**15-19**	0%	0%	0%	0%	0%	0%	0%	0%
	**20-24**	3%	3%	0%	0%	0%	0%	0%	0%
	**25-29**	1%	7%	5%	0%	1%	0%	0%	0%
	**30-34**	0%	5%	8%	7%	8%	0%	0%	0%
	**35-39**	0%	1%	3%	3%	8%	5%	0%	0%
	**40-44**	0%	0%	3%	6%	3%	3%	1%	0%
	**45-49**	0%	0%	0%	0%	3%	2%	3%	3%
	**50+**	0%	0%	0%	0%	2%	3%	3%	1%

Table 2: Assumed age distributions of partners in identified stable sero-discordant partnerships in the default model scenario, based on the Partners in Prevention Data (2, 3).

	Age	Pregnancy Incidence Rate (/100 woman-years)
Not Infected	15-24	30.38
	25-29	36.02
	30-34	22.66
	35-39	13.51
	40-44	4.94
	45+	0.00
HIV Infected	15-24	22.16
	25-29	12.42
	30-34	9.72
	35-39	7.79
	40-44	0.00
	45+	0.00

Table 3: Age-specific hazard of pregnancy. Source: Partners in Prevention data (2, 3). Note that the hazard of pregnancy among those on treatment is assumed to be equal to those uninfected of the same age.

Parameter	Value	Comments
Proportion of pregnancies preceded by period of "Pregnancy Intention"	80%	Illustrative value for overall comparison (see Discussion).
Duration of period of "Pregnancy Intention"	6 months.	Usage of contraception information is collected among couples (2, 3) and it was possible to estimate the time when some couples ceased using contraception (mid-point between last date of reported contraception use and first date of reported non-use of contraception). Among the women aged less than 25 years, the mean time to pregnancy was 6.1 months.
Increase in frequency of unprotected sex during period of "Pregnancy Intention".	4 additional unprotected sex acts per month.	Illustrative value for overall comparison (see Discussion). Calculated by assuming that overall chance of conception per unprotected sex act of 2% (aggregating across ovulation status) and the above median time to pregnancy, and the mean number of sex acts in couples at other times.

Table 4: Parameters specifying the nature of the period of "pregnancy intention" that precedes a pregnancy event in a set proportion of pregnancies and is characterized by an increase in the frequency of unprotected sex within the stable partnership for a fixed period of time.

**Man is infected**			
	**CD4 cell count category**		
**Man's age**	**200-350**	**350-500**	**500+**
**15-19**	27%	31%	42%
**20-24**	27%	31%	42%
**25-29**	27%	31%	42%
**30-34**	27%	31%	42%
**35-39**	27%	31%	42%
**40-44**	27%	31%	42%
**45-49**	27%	31%	42%
**50+**	27%	31%	42%

**Woman is infected**			
	**CD4 cell count category**		
**Woman's age**	**200-350**	**350-500**	**500+**
**15-19**	0%	50%	50%
**20-24**	24%	30%	46%
**25-29**	22%	34%	45%
**30-34**	26%	30%	45%
**35-39**	30%	21%	49%
**40-44**	30%	35%	35%
**45-49**	33%	42%	25%
**50+**	27%	55%	18%

Table 5: Distribution of CD4 cell count category of the HIV-infected partner at the moment that identified stable sero-discordant enters care. Source: Partners in Prevention Data (2, 3). Note, for infected men, due to small numbers, it was assumed that the distribution did not vary over age-groups.

Number of unprotected sex acts per month	Fraction
0-1	64%
1-2	14%
2-3	9%
3-4	4%
4-5	3%
5-6	2%
6-7	1%
7-9	1%
9-11	1%
11+	2%

Table 6: Assumed distribution of the frequency of unprotected sex within stable sero-discordant couple. Source: Partners in Prevention Data (2, 3).
This is based on the average of reported unprotected coital frequency in past months for each couple take at each of five six-monthly visits over two years. This distribution was found not to significantly interact with demographic variables and so this distribution was assumed to be the same for all partnerships. Partnerships are assigned to a category at the beginning of the simulation, and the number of sex acts each month is sampled randomly from within the bounds given by that category (under a uniform assumption and with the maximum number of sex acts per month equal to 30).

		Infectiousness Rate Ratio*	Mean Years In Category
**CD4 cell count if not treated**	**500+**	1.00	2.4
	**350-500**	1.00	2.4
	**200-350**	1.59	4.6
	**<200**	4.99	2.6
**If On ART**	-	0.08	n/a

Table 7: Representation of the course of HIV infection in the model. *The Infectiousness Rate Ratio is from Donnell *et al.* (11), and the ratio is given relative to an individual with CD4 cell count above 350 (in Donnell *et al*., there is no substantial difference between transmission of those with CD4 cell counts 350-500 or 500+ so these categories were combined here). The mean years spent in each CD4 cell count category is from a pooled-analysis of African observational cohort studies (12). Note that for individuals with CD4 cell counts below 200, the mean years in category parameter can be interpreted as mean survival time.

**Any external parnter over two years of follow-up (%)**		
	None	Any
Woman	92	8
Man	79	21

Table 8: The proportion of uninfected partners in identified stable sero-discordant partnerships reporting sex in the last month with another partner in any of the five six-monthly visits recorded over two years. No significant interactions with respect to age were found and so individuals were assigned to categories that ever or never had extra-marital partners in a manner that was independent of their age. There is a further assumption that, due to under-reporting (social desirability biases (13)) and the particular conditions of the trial (including intensive observation and provision of counselling services), the proportion of women reporting sex with another partner in the two years of the trial may be less than the proportion of women ever having sex with another partner over the full duration of the main stable partnership. The ratio between those two proportions is denoted γ (defined as the ratio of the assumed proportion of women that ever had sex with another partner compared to the proportion of women reporting sex with another partner in the two years of the study) and is fit through a systematic comparison of the model output and the trial data (see Table 13).

**Point prevalence of having an external partner**					
**Month**	**0**	**6**	**12**	**18**	**24**
**Women:**	0.00	0.03	0.03	0.03	0.02
**Men:**	0.02	0.09	0.11	0.09	0.05

Table 9: Point prevalence of having other sexual partner by time since enrolment in study in the Partners in Prevention trial (2, 3). Extern al partnerships were assumed to each last six months (in the data, the mean rate of acquiring new partners ranges between 4.4 months and 7.5 months depending on whether reports taken every 3 or 6 months are used) and in the absence of any clear trend between the probability of having an external partner and age, it is assumed that these rates apply for all ages (Chi-Squared tests: p=0.663 for women and p=0.856 for men). The number of sex acts per month in external partnerships is set equal to 3 for all partnerships and this is based on the reports from men and women at the final interview, two years after enrolment into the trial (earlier reports indicate a rate of unprotected sex close to zero, which is incompatible with the high rate of unlinked incident infections and is likely attributable to strong social desirability biases).

**Demographic Group**	**HIV Prevalence**
South African men 25-49	23.7%
South African women 20-34	32.7%

Table 10: Assumed prevalence among extra-marital partners of women (assumed to be South African men aged 25-49 years) and men (assumed to be South African women aged 20-34 years). Source: HSRC 2008 survey, estimates of HIV prevalence among 'most at risk groups' (5).

	First Year Mortality (%/year)	*Subsequent Year Mortality (%/year)*	*Source*	*First Year Drop-out (%/year)*	*Subsequent Year Drop-out (%/year)*
**CD4 cell count at ART Initiation**					
**>500**	1.3	1.3	(7)	10	5
**350-500**	2.5	1.3	(7)	10	5
**200-350**	5	2.5	(8)	10	5
**<200**	10	5	(9)	10	5

Table 11: Mortality rates and assumed drop-out rates on ART as a function of CD4 cell count at treatment initiation. The process of drop-out is modelled by an individual reverting to the CD4 cell count at which their treatment was initiated and then progressing as an untreated individual without the possibility of restarting treatment.

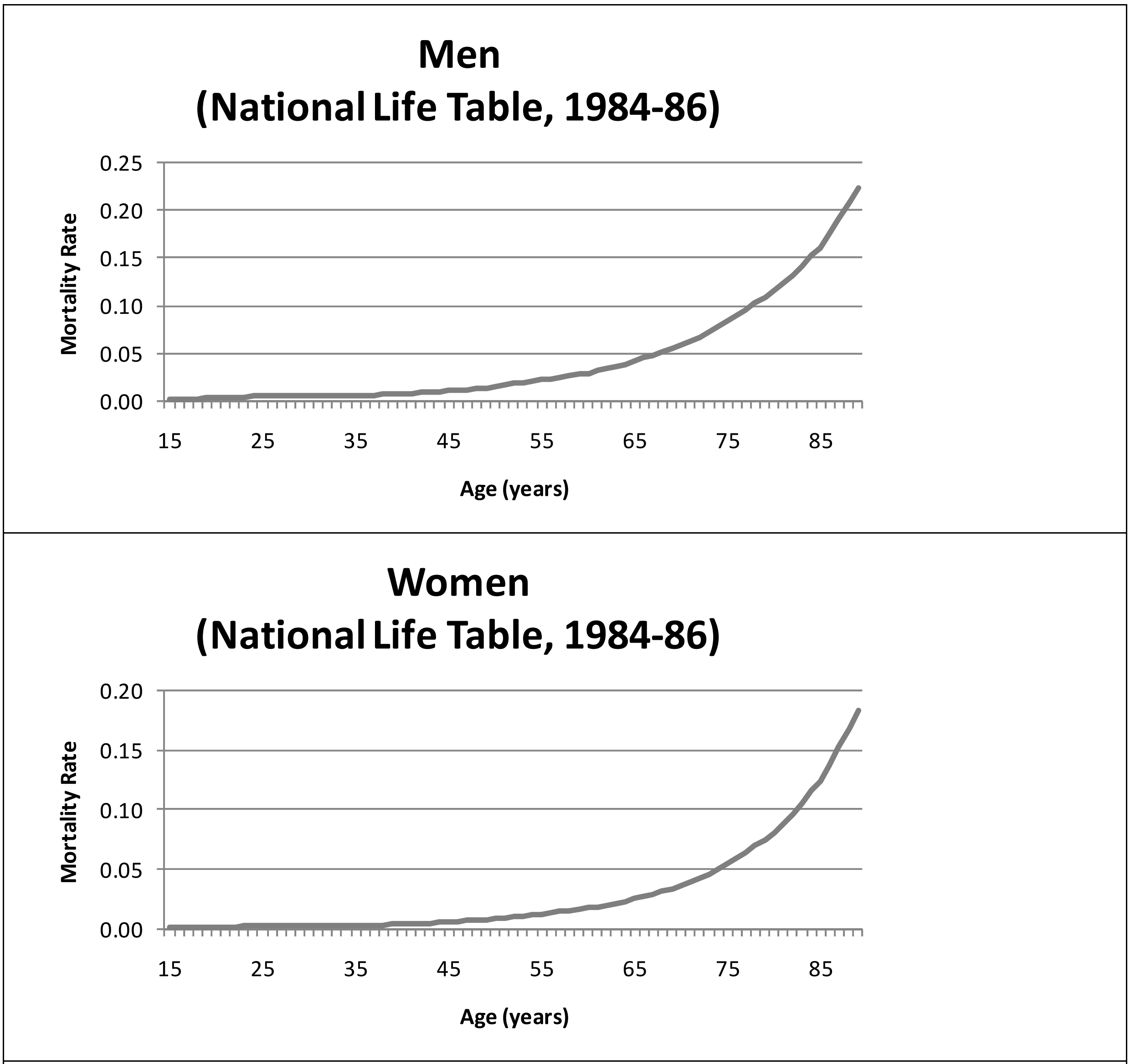

Figure 1: Assumed background (non-HIV) mortality rates for each member of the stable sero-discordant couple. The assumed rates are the result of fitting a function of the form $y = \alpha e^{\beta x}$ to the estimates in an original analysis by Dorrington *et al.* (14). Parameters are ($\alpha = 0.0013$, $\beta = 0.0689$) for men and ($\alpha = 0.0007$, $\beta = 0.0720$) for women (where y is the mortality rate and x is the exact age). Survival probabilities for each member of the partnerships are modelled independently.

Status	Utility Weights
Uninfected	1.0
HIV infected: CD4 cell count 500+	0.94
HIV infected: CD4 cell count 350-500	0.94
HIV infected: CD4 cell count 200-350	0.82
HIV infected: CD4 cell count below 200	0.70
On ART: First year	0.82
On ART: Subsequent years	0.94

Table 12: Assumed utility-weightings for each health state in the model. These weights are based on the those from a pooled analysis of weights by Tengs and Lin (2002) (15) that assigned a weight of 0.70 for AIDS, 0.82 for symptomatic HIV infection and 0.94 for asymptomatic HIV infection.

Calculation of Transmission Probability within a Stable Partnership

The probability of transmission within a stable sero-discordant couple each month (p) is modelled as:

$$p = 1 - \left\{ \left(1 - \beta_{C,A,S,P,Q}\right)^{n(1-\theta)} \left(1 - (1-\varepsilon)\beta_{C,A,S,P,Q}\right)^{n\theta} \right\}$$

Where $\beta_{C,A,S,P,Q}$ is the chance of HIV transmission per sex act with a partner with a CD4 cell count category *C*, ART-status *A*, sex *S*, pregnancy status of the woman *P* and circumcision status of the man in the partnership *Q*; n is the number of unprotected sex acts that month in the couple (Table 3) ; θ is the proportion of sex acts which are potentially protected by PrEP (denoted 'adherence'); and, ε is the 'intrinsic efficacy' of PrEP (defined as the reduction in the chance of transmission per sex act in which it is used and its full effects are accrued).

The chance of transmission per sex act is specified as:

$$\beta_{C,A,S,P} = \pi_C^C \pi_A^A \pi_S^S \pi_P^P \pi_Q^Q \beta_0$$

where β_0 is the "basic" chance of transmission per sex act from woman-to-man, when the infected partner is not on ART, has a CD4 count above 500, the woman is not pregnant and the man is not circumcised.

The terms π_C^C , π_A^A , π_S^S , π_P^P and π_Q^Q determine the relative change in the per sex act chance of transmission according to the CD4 count of the infected partner (Table 7), the ART-status of the partner ($\pi_0^A = 1$ for not on ART and $\pi_1^A = 0.08$ for on ART (Table 7)) , the sex of the partner ($\pi_1^S = 1$ for woman-to-man and π_2^S for man-to-woman (evaluated below)), the pregnancy status of each partner ($\pi_0^P = 1$ for neither partner pregnant, $\pi_1^P = 2.25$ if the uninfected partner is pregnant and $\pi_2^P = 2.18$ if the infected partner is pregnant (16)) and the circumcision status of the man ($\pi_2^Q = 0.35$ if uninfected male partner is circumcised and $\pi_1^Q = 1$ otherwise), respectively.

Calculation of Transmission Probability from an External Source

Uninfected individuals are divided into those that ever form external partnerships and those that do not (Table 8). Among those that do, a time-dependent rate of entering and exiting partnerships is applied such that the observed point prevalence of having at least one external partner is exactly reproduced (Table 9). It is assumed that those having external partners have at most one at each time, and that the minimum effective duration of each external partnership is 6 months (Table 9). Those external partners are not tracked explicitly but instead the infection status (infected or not infected) of an external partner is assigned (Table 10) at the initiation of an external partnership. If the external partner is infected, the probability of transmission each month is:

$$p_x = 1 - \left\{ \left(1 - \alpha_{S,P,Q}\right)^{n_x(1-\theta)} \left(1 - (1-\varepsilon)\alpha_{S,P,Q}\right)^{n_x \theta} \right\}$$

Where $\alpha_{S,P,Q}$ is the chance of HIV transmission per sex act with an external partner of sex S, where the pregnancy status of the infected partner is P and the circumcision status of the man is *Q*; and n_x is the number of sex acts per month with an external partner.

$$\alpha_{S,P,Q} = \kappa_S^S \kappa_P^P \pi_Q^Q \alpha_0 \beta_0$$

Where α_0 is the relative chance of transmission per sex act with an infected external partner compared to the infected stable partner (due to the greater chance of external partners being in the highly infectious acute phase of infection and higher chance of having sexually transmitted infections that increase the chance of transmission (4, 17, 18)) and κ_S^S and κ_P^P specify the change in per act chance of transmission if the uninfected partner is a woman ($\kappa_1^S = 1$ for woman-to-man and κ_2^S for man-to-woman) and if the uninfected partner is currently pregnant ($\kappa_1^P = 1$ for not pregnant and $\kappa_1^P = 2.25$ if pregnant (16)) , respectively. (No account is explicitly made of the possibility of the external infected partner being currently pregnant).

Calculation of PrEP Effectiveness

The parameter for the functional effect size of PrEpf PrEP (E) (referred to only as 'effectiveness' in the main text) combines assumptions about the "intrinsic efficacy" of PrEP and levels of adherence to the regimen and is analogous to the estimates of that will be generated in PrEP clinical trials. For a set of 'intrinsic efficacy' (ε) and 'adherence' (θ) parameters is calculated as follows. It is intended as a guide only and approximates the estimate of trial-effectiveness that will come from the on-going clinical trial among stable sero-discordant couples that will include follow-up for approximately 2 years (with 3 unprotected sex acts per month and an average per sex-act transmission probability of 0.001 (17)).

$$E = 1 - \frac{1 - \left\{ (1-0.001)^{2*3*12*(1-\theta)} \left(1 - (1-\varepsilon)0.001\right)^{2*3*12*\theta} \right\}}{1 - (1-0.001)^{2*3*12*}}$$

The following were the values used to generate different levels of functional efficacy in analyses in the model. We have explored the sensitivity of results to these assumption (i.e., generating the same value of E with different values of ε and θ and found this not to have a strong influence).

E =0.3 (ε =0.6 and θ=0.5); E =0.8 (ε =0.9 and θ=0.9).

Fitted Parameter Values for the "Partners in Prevention" scenario

The model's output pattern of incidence over the first two years of the simulation (to men from their infected stable partner, to men from other sources, to women from their infected stable partner and to woman from other sources) was fit to the observed incidence rates in the Partners in Prevention HSV/HIV trial in South Africa (Figure 2). The source of infection was determined by viral sequencing: if the infection is acquired from the stable partner, the infection is 'linked' and otherwise is 'unlinked' (2, 3). The four parameters manipulated in the fitting process are shown in Table 12. Fitting the model to each of the measured incidence rates was performed in sequence; varying β_0 to match model and data estimates for the incidence rate among men from a stable partner, varying α_0 to match model and data estimates for the incidence rate among men from external partner, and varying κ_2^S and γ together to match model and data estimates for the incidence rate among women from external partners. In each case, the relation between the parameter and the sum-of-squares error comparing model and data incidence rates was examined using a grid search techniques over a pre-specified plausible domain for the parameter, and the parameter value corresponding to the minimum sum-of-squares was selected. The resulting parameter fits are given in Table 12.

Parameter	Meaning	Fitted Value	Tolerated Values
β_0	Transmission rate from an asymptomatic, non-pregnant woman to uncircumcised man.	0.0013	0.0001-0.0014 (19)
α_0	Relative infectiousness of external partners compared to stable partners with asymptomatic infection (due to differences in prevalence of acute infection and co-factoring STIs).	25	1-40 (4, 18)
κ_2^S	Relative susceptibility of women (compared to men) at exposures to infection from extra-marital partners (due to differences in prevalence of acute infection and co-factoring STIs).	0.5	0.5-2.0 (17, 20, 21)
γ	Ratio of the assumed proportion of women that actually ever had sex with another partner compared to the proportion of women reporting sex with another partner in the two years of the study.	2.0	1.0-3.0

Table 13: Fitted parameters for the default ('Partners in Prevention') model scenario.

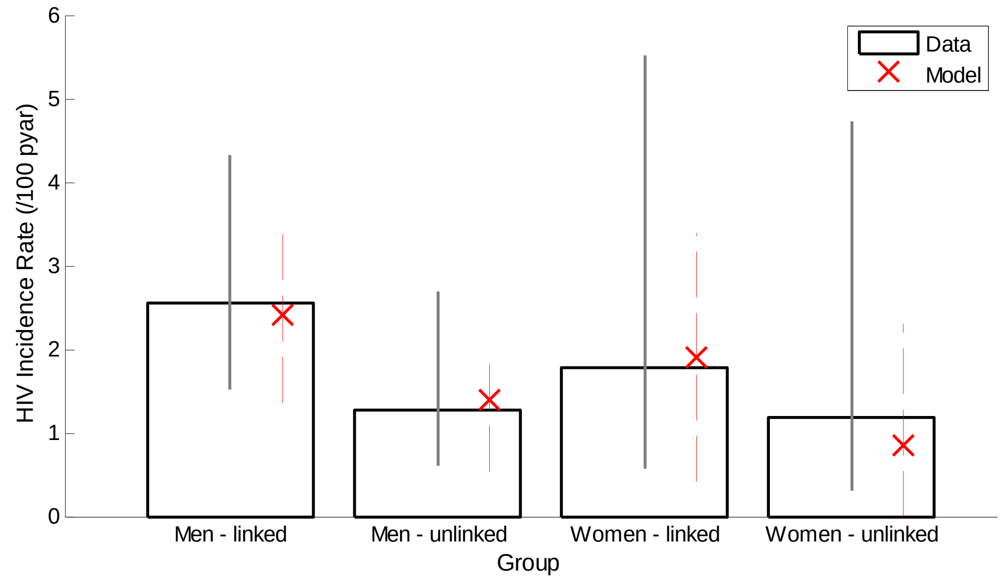

Figure 2: Comparison between empirical estimates of incidence (bars) and analogous simulated rates in the default model (red crosses) over the first two years of follow-up. The categories refer to the incidence rate among men from stable partner ('Men-linked'), incidence rate among men from external partner ('Men-unlinked), incidence rate among women from stable partner ('Women-linked') and incidence rate among women from stable partner ('Women-unlinked'). The whiskers on the bars correspond to the 95% confidence intervals of the point estimates and the whiskers on the point correspond to the range of estimate derived from repeated model simulations that include the same number of couples as the empirical estimates.

Sensitivity Analysis

Four alternative model scenarios were also constructed to explore how interventions can interact with different types of partnerships. Summary outcomes for each of these scenario (i.e. behaviours of couples) are given in Figures 3 and 4.

- *"Less Condom Use"*

This is the same as the default scenario (*PiP-SA*) with exception that condom use in the couple is set to zero (preserving the same reported rate of total sex). The distribution of unprotected sex in the couple is shown in Table 13.

Total number of sex acts per month	Fraction
0-1	5%
1-2	10%
2-3	14%
3-4	15%
4-5	11%
5-6	8%
6-7	6%
7-9	9%
9-11	6%
11+	15%

Table 14: Assumed distribution of the frequency of unprotected sex within stable sero-discordant couple under the "Less Condom Use" scenario. This is based on the average of reported total coital frequency in past month (those with condoms and those without combined) for each couple taken at each of five six-monthly visits over two years.

- *"More Extra Partners"*

This is the same as the default scenario ('Partners in Prevention') with the following two exceptions: (i) the proportion of men and women that can ever form an external partnership is equal to double what is estimated from the Partners in Prevention data (where having an external partner in the two years of the study is recorded and assumed to be equal to the proportion that ever have an extra-marital partner); and, (ii) the frequency of unprotected sex with external partners is double that estimated in the data (6 acts per month instead of 3 acts per month).

- *"More Infected Men"*

Whereas in the ('Partners in Prevention') scenario, in most couples it is the woman who is infected, in other studies (22) the proportions of men and woman that are infected in stable sero-discordant partnerships are more equal. In this alternative scenario, it is assumed that in 50% of stable sero-discordant partnerships the man is infected.

- *"More Typical Couples"*

In this scenario, all the above changes were implemented to some degree. The couples in this scenario used condoms 75% less often than reported in the baseline scenario (Table 14) and had more extramarital partners (50% more ever had extra-marital partners and the frequency of unprotected sex in external partners is double that reported) and a greater proportion (50%) included infected men. (Figure 3).

Unprotected sex acts per month	Fraction
0-1	9%
1-2	16%
2-3	17%
3-4	16%
4-5	8%
5-6	6%
6-7	7%
7-9	7%
9-11	5%
11+	9%

Table 15: Assumed distribution of the frequency of sex within stable sero-discordant couple under the “More Typical Couples” scenario. This is based on the average of reported total coital frequency in past month (those with condoms and those without combined) for each couple with an adjustment to allow for the model frequency of condom use in each couple to equal only 75% that reported in the data.

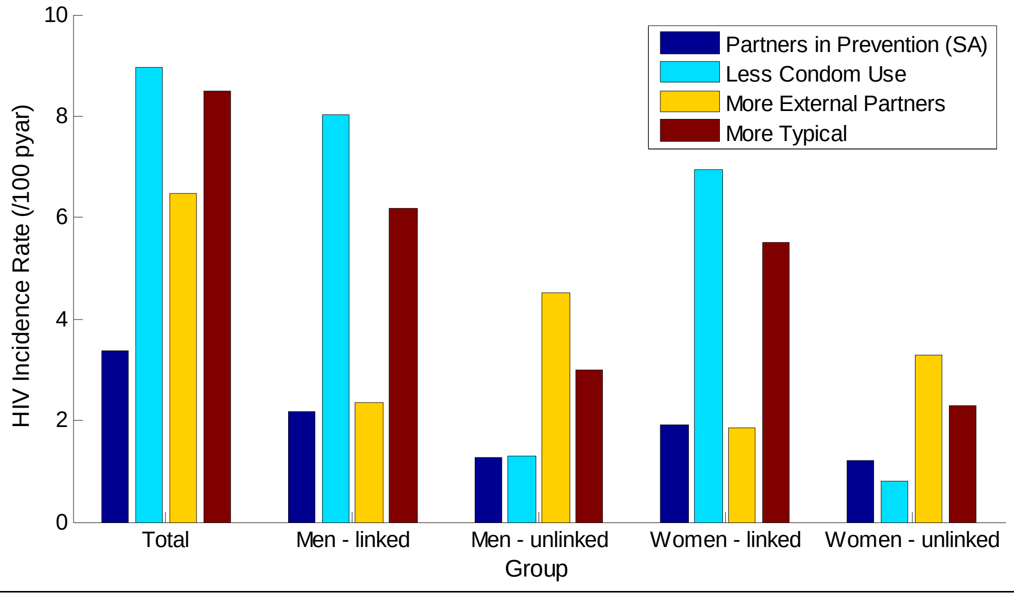

Figure 3: Model incidence rates over 2 years for the couples in each of the different scenarios. Note that the ‘More Infected Men’ scenario is not shown because it has the same incidence breakdown as the default (‘Partners in Prevention’) scenario. The grouping of the incident infections is the same as in Figure 2.

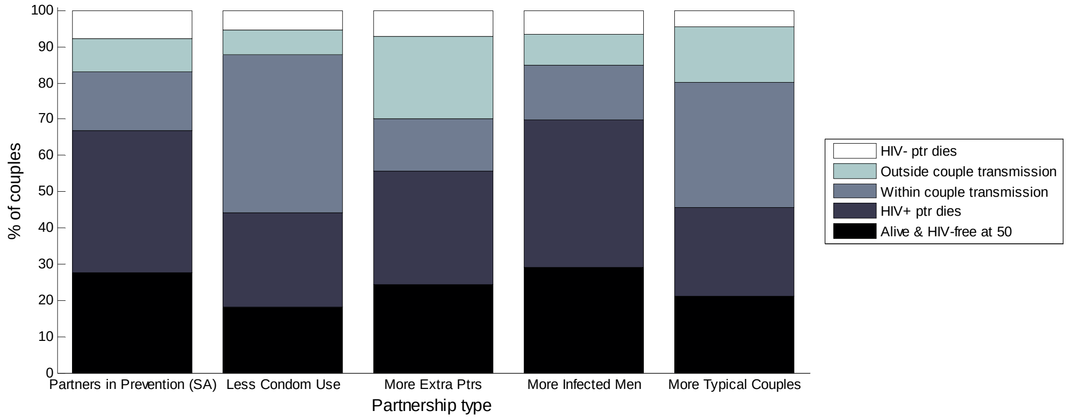

Figure 4: Outcomes of stable sero-discordant couples up to age 50 for each of the four scenarios are shown. Outcomes are categorized as either the couple reaching the age of 50 both ‘Alive and HIV Free’, or by the first event that prevents the couple reaching that state (The HIV-infected partner dies, there is a transmission event within the couple, there is a transmission event from an external source or the uninfected partner dies). It is assumed that ART is initiated at CD4 cell counts below 200 /µL and that no other interventions are in place.

References

1. South Africa National AIDS Council. The South Africa Antiretroviral Treatment Guidelines 2010 (http://www.doh.gov.za/docs/factsheets/guidelines/art.pdf, accessed 19-1-01)2010.
2. Lingappa JR, Kahle E, Mugo N, Mujugira A, Magaret A, Baeten J, et al. Characteristics of HIV-1 discordant couples enrolled in a trial of HSV-2 suppression to reduce HIV-1 transmission: the partners study. PLoS ONE. 2009;4(4):e5272.
3. Lingappa JR, Baeten JM, Wald A, Hughes JP, Thomas KK, Mujugira A, et al. Daily acyclovir for HIV-1 disease progression in people dually infected with HIV-1 and herpes simplex virus type 2: a randomised placebo-controlled trial. Lancet. 2010 Mar 6;375(9717):824-33.
4. Rottingen JA, Cameron DW, Garnett GP. A systematic review of the epidemiologic interactions between classic sexually transmitted diseases and HIV: how much really is known? Sex Transm Dis. 2001 Oct;28(10):579-97.
5. Shisana O, Rehle T, Simbayi L, Zuma K, Jooste S, Pillay-van-Wyk V, et al. South African National Prevalence, Incidence, Behaviour and Communication Survey, 2008. A Turning Tide Among Teenagers? Cape Town, South Africa: HSRC Press 2009.
6. Donnell D, Kiarie J, Thomas K, Baeten J, Lingappa J, Cohen C, et al. ART and Risk of Heterosexual HIV-1 Transmission in HIV-1 Serodiscordant African Couples: A Multinational Prospective Study [Paper #136]. 17th Conference on Retroviruses and Opportunistic Infections; San Francisco, CA2010.
7. Kitahata MM, Gange SJ, Abraham AG, Merriman B, Saag MS, Justice AC, et al. Effect of early versus deferred antiretroviral therapy for HIV on survival. N Engl J Med. 2009 Apr 30;360(18):1815-26.
8. Etard JF, Ndiaye I, Thierry-Mieg M, Gueye NF, Gueye PM, Laniece I, et al. Mortality and causes of death in adults receiving highly active antiretroviral therapy in Senegal: a 7-year cohort study. AIDS. 2006 May 12;20(8):1181-9.
9. Mahy M, Lewden C, Brinkhof MW, Dabis F, Tassie JM, Souteyrand Y, et al. Derivation of parameters used in Spectrum for eligibility for antiretroviral therapy and survival on antiretroviral therapy. Sex Transm Infect. 2010 Dec;86 Suppl 2:ii28-34.
10. Weiss HA, Halperin D, Bailey RC, Hayes RJ, Schmid G, Hankins CA. Male circumcision for HIV prevention: from evidence to action? AIDS. 2008 Mar 12;22(5):567-74.
11. Donnell D, Baeten JM, Kiarie J, Thomas KK, Stevens W, Cohen CR, et al. Heterosexual HIV-1 transmission after initiation of antiretroviral therapy: a prospective cohort analysis. Lancet. Jun 12;375(9731):2092-8.
12. The eligibility for ART in lower income countries collaboration. Duration from seroconversion to eligibility for antiretroviral therapy and from ART eligibility to death in adult HIV-infected patients from low and middle-income countries: collaborative analysis of prospective studies. Sex Transm Infect. 2008 August 1, 2008;84(Suppl_1):i31-6.
13. Gregson S, Zhuwau T, Ndlovu J, Nyamukapa CA. Methods to reduce social desirability bias in sex surveys in low-development settings: experience in Zimbabwe. Sex Transm Dis. 2002 Oct;29(10):568-75.
14. Dorrington R, Bradshaw D, Wegner T. Estimates of the Level and Shape of Mortality Rates in South Africa Around 1985 and 1990 Derived by Applying Indirect Demographic Techniques to Reported Deaths (http://www.mrc.ac.za/bod/1999report.pdf, accessed 1-11-10)1991.
15. Tengs TO, Lin TH. A meta-analysis of utility estimates for HIV/AIDS. Med Decis Making. 2002 Nov-Dec;22(6):475-81.

16. Mugo NR, Heffron R, Donnell D, Wald A, Were EO, Rees H, et al. Increased Risk of HIV-1 Transmission in Pregnancy: A Prospective Study among African HIV-1 Serodiscordant Couples.Submitted.
17. Wawer MJ, Gray RH, Sewankambo NK, Serwadda D, Li X, Laeyendecker O, et al. Rates of HIV-1 transmission per coital act, by stage of HIV-1 infection, in Rakai, Uganda. J Infect Dis. 2005 May 1;191(9):1403-9.
18. Hollingsworth TD, Anderson RM, Fraser C. HIV-1 transmission, by stage of infection. J Infect Dis. 2008 Sep 1;198(5):687-93.
19. Boily MC, Baggaley RF, Wang L, Masse B, White RG, Hayes RJ, et al. Heterosexual risk of HIV-1 infection per sexual act: systematic review and meta-analysis of observational studies. Lancet Infect Dis. 2009 Feb;9(2):118-29.
20. Padian NS, Shiboski SC, Jewell NP. Female-to-male transmission of human immunodeficiency virus. JAMA. 1991 Sep 25;266(12):1664-7.
21. Nicolosi A, Correa Leite ML, Musicco M, Arici C, Gavazzeni G, Lazzarin A. The efficiency of male-to-female and female-to-male sexual transmission of the human immunodeficiency virus: a study of 730 stable couples. Italian Study Group on HIV Heterosexual Transmission. Epidemiology. 1994 Nov;5(6):570-5.
22. Eyawo O, de Walque D, Ford N, Gakii G, Lester RT, Mills EJ. HIV status in discordant couples in sub-Saharan Africa: a systematic review and meta-analysis. Lancet Infect Dis. 2010 Nov;10(11):770-7.
